# Develop Your CORE_2_ for Career Flourishing: A Career Development Workshop for Hospitalists

**DOI:** 10.15766/mep_2374-8265.11387

**Published:** 2024-03-15

**Authors:** Ryan E. Nelson, Emily A. Mallin, Shannon K. Martin

**Affiliations:** 1 Instructor in Medicine, Department of Medicine, Harvard Medical School and Beth Israel Deaconess Medical Center; 2 Professor of Medicine, Department of Medicine, University of Arizona College of Medicine – Phoenix; 3 Associate Professor of Medicine, Department of Medicine, University of Chicago Pritzker School of Medicine

**Keywords:** Appreciative Inquiry, Career Development, Career Goals, Character Strengths, Flourishing, Positive Psychology, Professional Vision, Faculty Development, Mentoring/Coaching, Well-Being/Mental Health

## Abstract

**Introduction:**

Appreciative inquiry harnesses an individual's strengths to realize positive change, and a flourishing-focused mindset emphasizes engagement, social connectivity, and seeking meaningful work. Though the impact of these models on physician well-being and career planning has been evaluated in graduate medical education, their integration into career development initiatives for faculty has been limited. We designed a workshop to nurture hospitalist career development, based on our CORE_2_ conceptual framework (character strengths, overall vision, role assessment, explicit goals, and evaluation).

**Methods:**

We presented the workshop at the 2022 and 2023 Society of Hospital Medicine (SHM) annual conferences. This 1.5-hour workshop comprised four modules and three small-group activities designed to help participants identify their signature character strengths, draft a professional vision statement, prioritize professional roles, and develop SMART goals aligned with these roles.

**Results:**

At the 2023 SHM annual conference, 36 participants attended the workshop, and 32 (89%) completed pre- and postworkshop surveys. After workshop completion, participants' self-assessed familiarity with their signature character strengths, knowledge of evidence-based principles to develop SMART goals, and confidence in their ability to write a vision statement and SMART goals all increased significantly (*p* < .05).

**Discussion:**

This workshop provides a valuable framework for self-directed longitudinal career development and reflection. We build on prior curricula on educator identity formation by guiding participants from identity definition to professional vision development to professional role evaluation to aligned goal creation and iterative evaluation. Our workshop's principles are readily generalizable to clinician-educators across medical disciplines.

## Educational Objectives

By the end of this workshop, participants will be able to:

1. Describe CORE_2_, a five-step framework for building career success on a foundation of personal fulfillment.
2. Identify and reflect on their signature character strengths.
3. Draft a specific and motivational professional vision statement.
4. Develop professional SMART goals to jump-start their 1- and 5-year career plan.
5. Apply CORE_2_ to facilitate flourishing throughout longitudinal career development.

## Introduction

Early-career physicians and advanced practice providers (APPs) seeking to jump-start a career in academic medicine face numerous barriers to entry. This is especially true for hospitalists, a specialist group that has faced significant challenges with obtaining academic mentorship, resulting in reduced scholarly productivity and promotion.^1–3^ This deficit is due in part to the rapid expansion of hospital medicine with a resultant imbalance between early-career mentees and available mid- to late-career mentors.^4,5^

Current research highlights important external barriers to academic career development, including identifying a local professional niche; securing funding, protected time, and personnel for scholarly activities; and connecting with like-minded mentors, coaches, and sponsors.^6^ Two equally important but underrecognized barriers are internal: fixation on a problem-solving mindset and misconception of career flourishing.

In clinical practice, early-career physicians and APPs often adopt a problem-solving approach and ask, “What is going wrong?” Appreciative inquiry advocates the alternative mindset: “What is going well?” Applying appreciative inquiry to career development encourages individuals to leverage their existing strengths to realize self-efficacy and envision positive future change.^7^ Flourishing is a well-described conception of individual well-being that comprises five elements: positive emotions, engagement in activities, strong relationships with peers, involvement in meaningful work, and personal accomplishment.^8^ A common misconception in professional development is that career success is a prerequisite for flourishing. Yet positive psychology research supports the opposite relationship: Experiencing flourishing helps build the foundation for realizing career success.^9^ Connecting these themes, appreciative inquiry identifies then harnesses one's character strengths to facilitate flourishing, which propels career success.^8,10,11^

While these concepts have gained traction in recent graduate medical education literature,^6,7,12,13^ their synthesis into faculty career development workshops has been limited. Previous faculty development workshops have focused on professional identity formation in academic medicine, citing three major influences on its development: context (feeling supported), roles (feeling engaged), and agency (feeling empowered).^14,15^ Identity embodies one's self-understanding, self-presentation, and self-recognition and represents a professional starting point.^16^ Conversely, professional vision orients one's career path and describes the ideal professional end point: “beginning with the end in mind.”^17^ We found limited medical education literature on professional vision formation for individuals, as most studies focus on developing organizational vision. Furthermore, we did not find any studies in the medical education or higher education literature specifically linking an academician's professional vision formation with the creation of role-oriented career development goals.

**Figure 1.**
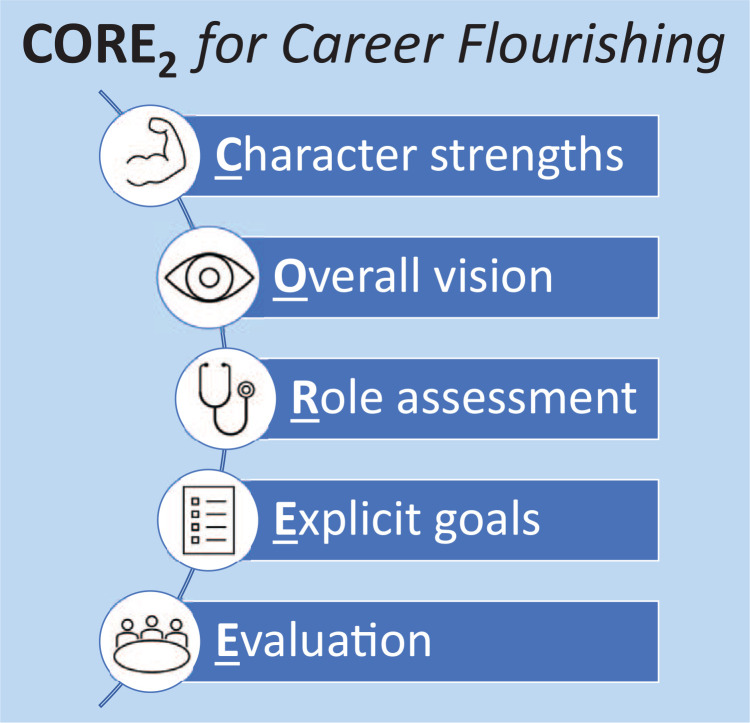
The CORE_2_ (character strengths, overall vision, role assessment, explicit goals, and evaluation) conceptual framework. CORE_2_ is a five-step, sequential framework for building career success on a foundation of personal fulfillment.

Recognizing these gaps, we felt that clinician-educators, especially academic hospitalists, would benefit from an evidence-based framework for career planning that emphasizes character strengths and flourishing as the catalysts for focusing professional vision and developing career goals. Based on this premise, we designed, implemented, and evaluated Develop Your CORE_2_ for Career Flourishing—a faculty development workshop to help hospitalists build career success on a foundation of personal fulfillment. Our workshop builds on our previously published sequential career development model CORE_2_, which stands for character strengths, overall vision, role assessment, explicit goals, and evaluation (Figure 1).^18^

Our CORE_2_ model is grounded in positive psychology and industrial and organizational psychology research.^18^ The first step of CORE_2_ is recognition of one's character strengths through appreciative inquiry. Character strengths are “positive traits, intrinsic to our personalities, that impact how we think, feel, and behave.”^10^ Signature character strengths reflect one's core values and basic identity and facilitate one's sense of flourishing.^8,11^ The second step is creation of an overall professional vision statement that draws on one's signature character strengths and helps define career vision. An effective vision statement incorporates seven attributes: conciseness, clarity, future orientation, stability, challenge, abstractness, and ability to inspire.^19^ The vision statement clarifies the third step of CORE_2_, selection of the most meaningful current and desired future professional roles that will realize the vision, such as scholar, peer mentor, or director of quality improvement. The fourth step is development of SMART (specific, measurable, achievable, relevant, and time-bound) goals, aligned with professional roles and organized into a 1- and 5-year plan, to provide clear action items and deadlines for deliverables, such as academic scholarship. The fifth and final step is evaluation of this process through reflection and revision of one's vision statement and recommitment to professional roles and SMART goals. This iterative appraisal highlights successes and renews one's sense of career flourishing.

The developers of this resource are academic hospitalists with interests and expertise in professional development. All have experience and training in medical education and curriculum development, and one (Emily A. Mallin) is a national leader in faculty development in hospital medicine via leadership of the Academic Hospitalist Academy, a professional development work cosponsored by the Society of Hospital Medicine (SHM), the Society of General Internal Medicine, and the Association of Chiefs & Leaders of General Internal Medicine.

## Methods

### Curriculum Design and Educational Strategies

To introduce, justify, and allow participants to apply the CORE_2_ framework, we created a 1.5-hour workshop comprising four didactic modules and three small-group breakout activities (Appendix A). Our rationale behind selecting this format was to introduce and ground the framework as based on positive psychology and to allow appropriate time to work through each component of the CORE_2_ framework. We designed the workshop to utilize didactic instruction, discussion, reflection, and application to one's own career and personal circumstances. We considered that while some framework elements, such as SMART goals, might be familiar to participants, others, including professional vision statements and strengths inventories, could be new concepts; thus, we wished to design an interactive workshop that gave participants time and space for both learning and applying potentially new constructs.

We began the workshop by highlighting the importance and relevance of career development in academic medicine, outlining the general time frame, and encouraging audience engagement during small-group activities. Module 1 introduced the concepts of character strengths and flourishing as the catalysts for career success. We advocated for a change from focusing on weaknesses to practicing appreciative inquiry, the mobilization of character strengths to promote positive future change.^7^ We defined and reviewed character strengths and virtues and provided examples. We then introduced the Values in Action (VIA) Character Strength Survey as a publicly available instrument with validity evidence for measuring one's unique character strength profile.^20^ Next, we reviewed the concept of flourishing and introduced the framework's central tenet: Rather than career success being the prerequisite to flourishing, career flourishing is the prerequisite and primer for career success.^8^

In breakout activity 1, participants reviewed the character strengths and virtues handout (Appendix B). We asked them to star the character strengths that resonated with their core values and then identify their top-five signature strengths. They recorded these signature strengths and reflections on a worksheet (Appendix C). We provided participants with a link to take the VIA Character Strengths Survey as an optional exercise after the workshop.^20^ Following this self-reflection exercise, we encouraged participants to share insights with their small groups.

Module 2 began with the description of a professional vision statement, which we argued should be informed by one's signature character strengths to potentiate career flourishing. We then reviewed the seven evidence-based components of an impactful vision statement.^21,22^ We provided examples from prominent business and world leaders to demonstrate the value of a vision statement^23^ and then shared our own as clinician-educator models. For breakout activity 2, we invited participants to compose a rough draft of their vision statement on the worksheet (Appendix C) and share the drafts in small groups for feedback.

**Figure 2.**
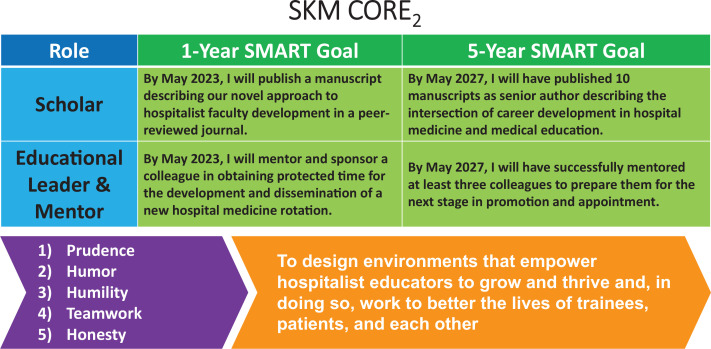
Completed CORE_2_ (character strengths, overall vision, role assessment, explicit goals, and evaluation) framework. This graphic illustrates the completed CORE_2_ framework for one of the authors (Shannon K. Martin [SKM]). Character strengths (bottom left) and a professional vision statement (bottom right) form the necessary foundation for professional role assessment (top left) and then for 1- and 5-year SMART (specific, measurable, achievable, relevant, and time-bound) goal development (top right).

Module 3 focused on professional roles and goals. We provided a table with example professional roles and recommended using one's vision statement to illuminate the most important professional roles, as this practice would inform prioritization of career goals. Next, we reviewed the SMART goal framework for writing 1- and 5-year career goals aligned with professional roles. To illustrate the synthesis of character strengths, vision statement, professional roles, and aligned career goals, we included graphics depicting our CORE_2_ drafts (Figure 2), explaining how each step informed our career development. We transitioned to breakout activity 3, prompting participants to reflect on their current and future desired professional roles and record two to three high-priority roles on their worksheet (Appendix C). We invited them to compose a 1- and 5-year SMART career goal for each chosen professional role and reflect on their roles and goals in small groups.

We concluded with module 4, which underscored the habit of evaluation as the key final step in the CORE_2_ framework. We recommended that participants reflect on their vision statement biannually and recommit to their professional roles and corresponding SMART goals monthly and suggested tips for accountability, such as displaying a graphic representation of their CORE_2_ framework in a prominent location and reviewing it with a trusted mentor or coach. We provided a final review of the CORE_2_ framework, shared a graphic template for participants to illustrate their CORE_2_ drafts (Appendix D), and invited further questions.

### Implementation of the Curriculum

We utilized PowerPoint slides to teach didactic modules and to define the assignments and time frames for each small-group activity (Appendix A). Each participant received a printed character strengths and virtues handout (Appendix B) and a worksheet (Appendix C) to facilitate the small-group activities and to reference as they developed and refined their CORE_2_ frameworks. We required the following resources to run the workshop successfully: a large projector screen, a clicker for the slide deck, lavalier microphones, preprinted handouts and worksheets, and tables to facilitate small-group activities. We developed a facilitator's guide (Appendix E) to aid instructors leading the didactic modules and breakout activities.

We piloted Develop Your CORE_2_ for Career Flourishing as an in-person workshop at the SHM annual conference in 2022. After a successful pilot with excellent speaker scores and feedback, we presented the workshop again at the SHM annual conference in 2023, where we obtained evaluation data from participants. The University of Chicago Institutional Review Board granted educational exemption for evaluating this curriculum.

### Evaluation of the Curriculum

We evaluated the curriculum using pre- and postsession surveys (Appendices F and G, respectively). We developed all survey items through consensus-driven, iterative review among the authors. The survey included nominal, ordinal (5-point Likert scales), dichotomous, and open-ended questions. In the presurvey, we collected basic demographic information regarding participants' level of training. In both surveys, we included the same 5-point Likert scale items, using construct-specific response anchors, to evaluate changes in participants' familiarity with their signature character strengths, knowledge of evidence-based principles to develop SMART goals, confidence in writing a vision statement and SMART career goals, and perspective on a vision statement's importance for career development (1 = *not at all confident*, 5 = *extremely confident*). In the postsurvey, we used a 5-point Likert scale to evaluate participants' perception of the workshop's importance to their career development (1 = *not at all important,* 5 = *extremely important*), asked them if they would recommend the workshop to a colleague (yes/no), and requested additional feedback.

## Results

Since April 2022, over 50 hospitalist physicians, APPs, and trainees interested in hospital medicine have completed the workshop across two sessions at SHM's annual conference. We evaluated the workshop during the 2023 SHM annual conference, during which 32 out of 36 participants (89% response rate) completed the pre- and postworkshop surveys. Participants included 26 MDs/DOs, three APPs, and three resident physicians. The average number of years in practice for MDs/DOs and APPs was 5.75, with a range of 1–24 years. Residents were in postgraduate years 2–4. We analyzed responses to pre- and postworkshop knowledge and attitudinal questions using the Wilcoxon signed rank test.

**Figure 3.**
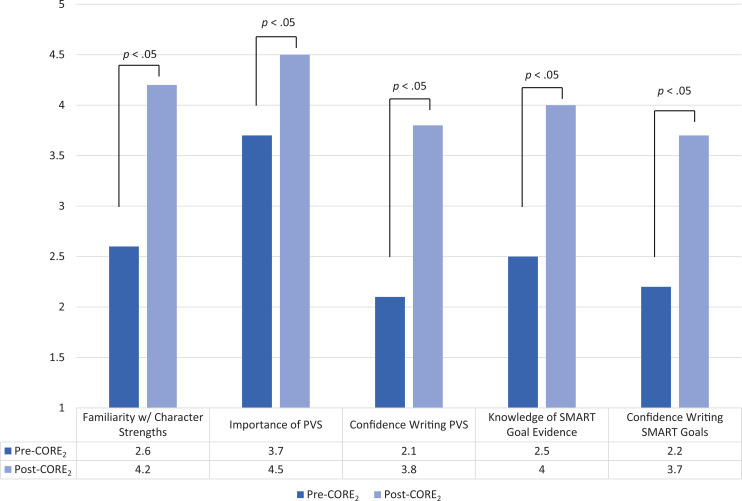
Pre- and postworkshop survey data. After completing the workshop, participants' self-assessed familiarity with their signature character strengths, knowledge of evidence-based principles to develop SMART (specific, measurable, achievable, relevant, and time-bound) goals, confidence in their ability to write a professional vision statement (PVS) and SMART career goals, and valuation of writing a PVS towards career development all increased significantly.

In preworkshop surveys, most participants reported themselves as being not at all or slightly confident in writing a vision statement (88%) and SMART career goals (88%) and not at all or slightly familiar with their signature character strengths (75%). After completing the workshop, participants' self-assessed familiarity with their signature character strengths, knowledge of evidence-based principles to develop SMART goals, confidence in their ability to write a vision statement and SMART career goals, and valuation of writing a vision statement towards career development all increased significantly (*p* < .05; Figure 3). Most participants (91%) reported that their workshop experience was either very or extremely important to their career development, and all (100%) would recommend the workshop to a colleague.

Participants described the workshop as “a great catalyst [to get] the gears started” for planning their academic career development. They valued the “concise strategies,” dedicated “time and space to think about professional vision,” and encouragement to “put thoughts on paper to make goals more tangible.” They appreciated the interactive breakout sessions and “loved the discussion and help from [their] tables.” For future workshops, they recommended matching a “facilitator to each group to evaluate each person's statements/goals to provide more guidance/reflections” and including example roles and goals from a “clinical/non-academic perspective.”

## Discussion

We created a workshop to provide hospitalists with a sequential framework for building their professional careers on a foundation of personal fulfillment. Our workshop increased participants' familiarity with their signature character strengths, which helped clarify professional values and identity. Participants recognized the importance of developing a professional vision for their career development and reported increased confidence in transforming this vision into a formal vision statement. Translating a vision statement into action necessitates concrete goals aligned with professional roles, and participants reported improved knowledge of SMART goal elements and confidence in writing them. They valued the interactive discussions in small groups and the protected time for self-reflection on their professional growth. This single-session workshop was valuable to participants' career development, and all would recommend enrollment to their colleagues.

Our workshop builds on prior research and faculty development seminars on clinician-educator identity formation by guiding participants along the pathway from identity recognition to professional vision realization to professional role evaluation to aligned goal creation.^14,15^ The workshop is innovative in that its CORE_2_ conceptual framework synthesizes evidence-based principles from the fields of positive psychology, industrial and organizational psychology, and career coaching.^8–11,19–23^ Importantly, our emphasis on adopting appreciative inquiry and a flourishing-focused mindset prioritizes personal empowerment and well-being as essential (yet often overlooked) skills for developing a successful career in academic medicine.^6,7,12,13^

Career development planning is a vital component of mentorship, as it provides structure and tangible goals to facilitate longitudinal growth for the mentee. Our workshop provides a self-directed framework, requiring minimal resources, that any faculty member can utilize to jump-start their academic career planning and continuously reflect on their progress even in the absence of consistent career mentorship. Our workshop is especially valuable for academic hospitalists—specialists with reduced access to career mentors, resulting in decreased scholarly productivity and promotions.^1–3^

Though we implemented our workshop among our academic hospitalist peers, we believe its principles are generalizable across medical disciplines. Our overall goal is to make this workshop readily accessible to the wider academic medicine community to provide clinician-educators and medical education leaders with an evidence-based approach to longitudinal career development that sustains well-being and may help combat burnout.

We pilot tested the workshop in person at SHM's 2022 annual conference. Following its success there, we delivered it in person again at the SHM 2023 annual conference, where we obtained evaluation data. In the interim time period, we were additionally invited to present the workshop in a virtual hour-long format at an academy of interdisciplinary educators at one of our home institutions and as an hour-long webinar via a national professional development organization. Comparing these experiences, we strongly recommend delivering the workshop in person to maximize participants' engagement in the small-group sessions where they apply the didactic material to develop their CORE_2_ frameworks. However, the virtual format does present advantages in ease of delivery and still facilitates discussion, albeit more limited. Incorporating feedback from our pilot 2022 workshops, we have condensed the didactic portions and expanded the time for each small-group session, added our own vision statements (Appendix A, slide 19) and personalized CORE_2_ graphics (Appendix D) to provide examples for participants, and created an easy-listening playlist to enhance the environment during self-reflection and writing activities. Based on the most recent 2023 workshop feedback, we suggest having one facilitator per small-group table (as resources allow) to enhance participant engagement by designating a point person for questions and feedback on vision statements and SMART career goal drafts.

One challenge we have faced is maintaining mastery of the positive psychology and industrial and organizational psychology principles supporting our CORE_2_ framework. Based on our experience, facilitators should regularly review the literature cited within this publication to maximize the impact of the workshop. Furthermore, each facilitator should take time to develop, reflect upon, and evaluate their own CORE_2_ framework prior to leading others through the workshop as facilitator examples and reflections out loud are particularly helpful to the audience.

Our pre- and postworkshop surveys assessed attitudinal and knowledge-based outcomes but did not directly measure skill-based outcomes such as completing a vision statement or finalizing SMART goals. We do not have long-term follow-up with participants to measure their adoption and adherence to the CORE_2_ framework or its effect on professional growth, though comments support the workshop's positive influence on career development. Lastly, our participants during the evaluated workshop (2023 SHM national conference) were limited to academic hospitalists.

We are planning a multi-institutional collaboration to measure the long-term impact of CORE_2_ on clinician-educators' scholarly productivity, leadership involvement, academic promotion, retention, and sense of career flourishing and burnout.

## Appendices


Modules 1-4.pptxCharacter Strengths and Virtues Handout.docxParticipant Worksheet.docxGraphic Template.pptxFacilitator Guide.docxPresurvey.docxPostsurvey.docx
*All appendices are peer reviewed as integral parts of the Original Publication.*
