# Supplementary material for: Develop Your CORE2 for Career Flourishing: A Career Development Workshop for Hospitalists
Source: MedEdPORTAL. 2024 Mar 15;20:11387. doi: 10.15766/mep_2374-8265.11387 (PMC10940547; doi:10.15766/mep_2374-8265.11387)
Supplement: Supplementary file 1 — Modules 1-4.pptxCharacter Strengths and Virtues Handout.docxParticipant Worksheet.docxGraphic Template.pptxFacilitator Guide.docxPresurvey.docxPostsurvey.docx [file mep_2374-8265.11387-s001.zip › B. Character Strengths and Virtues Handout.docx]

| **Virtue** | **Character Strengths**^1,2^ |
| --- | --- |
| Wisdom | Creativity |
|  | Curiosity |
|  | Judgement |
|  | Love of Learning |
|  | Perspective |
| Courage | Bravery |
|  | Honesty |
|  | Perseverance |
|  | Zest |
| Humanity | Kindness |
|  | Love |
|  | Social Intelligence |
| Justice | Fairness |
|  | Leadership |
|  | Teamwork |
| Temperance | Forgiveness |
|  | Humility |
|  | Prudence |
|  | Self-Regulation |
| Transcendence | Appreciation of Beauty and Excellence |
|  | Gratitude |
|  | Hope |
|  | Humor |
|  | Spirituality |

**Optional Exercise:**

Consider taking the VIA Character Strengths Survey after the workshop, available for free online:

- Go to the VIA Institute on Character website at <https://viacharacter.org>
- Click “Take the Free Survey”
- Under "Register to take a VIA survey," fill in your information and register for a free account
- Click "Begin the survey”

**References:**

1. Peterson C, Seligman MEP. *Character strengths and virtues: A handbook and classification*. Oxford University Press; 2004.
2. The VIA character strengths survey. VIA Institute on Character. Accessed January 19, 2022. <https://www.viacharacter.org/account/register>.
