# Supplementary material for: Develop Your CORE2 for Career Flourishing: A Career Development Workshop for Hospitalists
Source: MedEdPORTAL. 2024 Mar 15;20:11387. doi: 10.15766/mep_2374-8265.11387 (PMC10940547; doi:10.15766/mep_2374-8265.11387)
Supplement: Supplementary file 1 — Modules 1-4.pptxCharacter Strengths and Virtues Handout.docxParticipant Worksheet.docxGraphic Template.pptxFacilitator Guide.docxPresurvey.docxPostsurvey.docx [file mep_2374-8265.11387-s001.zip › C. Participant Worksheet.docx]

**Breakout Activity**  **#1: Evaluate Your Character Strengths**

- Review the Virtues and Character Strengths table on the handout.
- Star the character strengths that resonate with your core values.
- From your starred list, identify **5 signature strengths** and record these on your worksheet.
- Reflect on your signature strengths and share insights with your group.
- **Optional:** After the workshop, consider taking the VIA Character Strengths Survey, available for free online at [https://viacharacter.org](https://viacharacter.org/)

Record your unique **Signature Strengths** (Top-5 Character Strengths)

1. ______________________________ 4. ______________________________

2. ______________________________ 5. ______________________________

3. ______________________________

Reflections on my signature strengths: __________________________________________________________________________________________________________________________________________________________________________________________________________________________________________

**Breakout Activity #2: Draft your Professional Vision Statement (PVS)**

7 characteristics of an impactful PVS: **1) Conciseness, 2) Clarity, 3) Future Orientation,**

**4) Stability, 5) Challenge, 6) Abstractness, 7) Desirability & Ability to Inspire**

________________________________________________________________________________________________________________________________________________________________________________________________________________________________________________________________________________________________________________________

______________________________________________________________________________

______________________________________________________________________________

______________________________________________________________________________

______________________________________________________________________________

**Breakout Activity #3: Define Your Roles & Goals**

- Revisit your PVS for inspiration.
- Consider both professional and personal roles.
- Record 2-3 roles on the worksheet.
- Using the SMART framework, develop a 1- and 5-year goal for each role.

**SMART = S**pecific, **M**easurable, **A**ttainable, **R**elevant, **T**ime-bound

| Role: | 1-Year Goal: | 5-Year Goal: |
| --- | --- | --- |
|  |  |  |
|  |  |  |
|  |  |  |
