# Supplementary material for: Develop Your CORE2 for Career Flourishing: A Career Development Workshop for Hospitalists
Source: MedEdPORTAL. 2024 Mar 15;20:11387. doi: 10.15766/mep_2374-8265.11387 (PMC10940547; doi:10.15766/mep_2374-8265.11387)
Supplement: Supplementary file 1 — Modules 1-4.pptxCharacter Strengths and Virtues Handout.docxParticipant Worksheet.docxGraphic Template.pptxFacilitator Guide.docxPresurvey.docxPostsurvey.docx [file mep_2374-8265.11387-s001.zip › E. Facilitator Guide.docx]

**Overview**: Variations of this presentation have been given in a variety of settings, including informal small-group settings, large in-person workshops, webinars without participant interaction, and online sessions with some interaction. We have used 1-3 speakers in these settings and encourage no more than three speakers total. Speaker(s) should be able to easily serve as facilitators during small group discussions, as the breakout activities are intended to be participant-driven.

Due to the flexible design of the workshop that can be tailored to various educational venues, facilitator roles are adaptable to the forum and audience. This guide is intended for use with all types of sessions and will offer specific tips for facilitation specific to the most common types of workshop venues.

The general flow of the presentation, regardless of forum, alternates didactic and active learning. Three short didactic modules are each followed by breakout activities that include active reflection, individual work, and small group discussion. Report outs are not necessary, but one or two salient comments may be shared with the large group as it returns for the next didactic session. The presentation concludes in the larger group, and opportunity should be given to answer questions at the end.

Facilitators are available during breakout activities to respond to questions (see FAQ below) or may need to intervene by asking questions to prompt discussion when participants feel “stuck”, but in general, are available mostly to listen, take notes that might be valuable takeaways, and keep people on track with time.

Facilitators (especially if speakers) should be individuals familiar with the workshop content. We strongly suggest that facilitators complete all components of the workshop in advance for themselves, including:

- Character strengths assessment
- Professional (and personal, if desired) vision statement (Module 2, slide 19)
- Role assessment
- SMART goal development
- Graphic template (Module 3, slide 25-27, Appendix D)

We recommend customizing the slide deck by inserting facilitator information above for enhanced connection with the audience.

**Set up:** Slide deck customized as above for use either online or in person use with appropriate microphone (including one or two for audience if appropriate), camera, and pointer capabilities. Timekeepers for didactics and breakout activities should be determined in advance ( ~10 min per breakout activity).

**In person workshop:**

Organize seating into small groups of 4-6 participants, with ability to easily return to screen for didactics.

Each table should have hard copies of the following documents (1 per participant):

- Virtues and Character Strengths table (for use in breakout activity #1) – Appendix B
- CORE_2_ worksheet (for use in breakout activity #1-3) – Appendix C

**Online workshop:**

We recommend interactive meeting (preferred over webinar) when possible, speaker(s) with video on. We encourage all participants to also have video on for optimal engagement and feedback (depending on institutional culture). Electronic copies of (or links to) materials should be included in meeting invite. Links should also be made available in real time to participants in chat in main room and also in breakout rooms if possible. Organize breakout rooms in advance and assign participants at the appropriate times, either at random or in groups based on stated goals of the session. We encourage smaller groups when done online (3-4 participants per breakout). Participants should be able to easily go from main meeting space to the same breakout for all activities. All handouts should be shared in advance and made accessible through chat function at the start of the workshop. ***We strongly encourage the use of a support staff member or other non-speaker to assume this role.***

**Breakout Activity #1**: Evaluate your Character Strengths. This activity is cognitively “light” and should be easy (and fun!). Facilitators will orient participants to the two handouts for this session and instruct them to identify their strengths from the Character Strengths and Virtues Handout (Appendix B) and list them on the worksheet (Appendix C). Participants will not need too much reflection time and should be encouraged to interact with one another. Some prompts to consider (if needed) once everyone has written down strengths:

- “Any surprises or unusual strengths?”
- “How/why you did you choose those 5 strengths?”
- “How do or should your strengths inform your work to encourage flourishing?”

**Breakout Activity #2**: Draft your Professional Vision Statement. This activity is the most mentally challenging, and participants should be reminded that they should not expect to have completed a vision statement by the end of the workshop, both when introducing the activity and repeated after the breakout. A vision statement takes time for drafting and editing, so encourage individuals to at least have some key words or phrases to include and connect back to character strengths. The activity should begin with some individual reflection time (4-5 min), during which time we have used calm music (acoustic/instrumental) to enhance the climate for reflection. Invite participants to share what they have, even if incomplete. Offer positive reinforcement for anything that is accomplished during this time. This time is flexible, so if the group wants more time to think/write uninterrupted, please allow. If participants are clearly struggling after about 2 min, specific prompts could include:

- “Look again at your strengths and finish this sentence, ‘With this strength, I will be able to ___.’”
- “Think about what kind of impact you seek to have professionally. What do you want to be known for/as?”

**Breakout Activity #3**: Define your Roles and Goals. This activity will require the most time management to get through everything smoothly, so we recommend using a timekeeper.

- **First 2-3 min**: Have participants start by listing roles (not necessarily titles, but professional roles) they currently hold, which should be straightforward. Then, they will consider roles they would like to have in the future – a job/position, a role they would like to develop, etc. If current and desired roles are the same, great, this individual may be flourishing! For some participants who haven’t considered this before, this exercise could take some time, but in general, this part of the activity should be completed quickly to provide more time for drafting goals. As above, this is a good time for calm music.
- **Next 2-3 min**: Ask participants to pick 1-2 goals for each desired role and to draft a 1- and 5-year SMART goal for each.
- **Final 4-6 min**: Invite participants to briefly state one desired role and one or both SMART goals, encouraging participation even if incomplete. Offer positive reinforcement for anything that is accomplished during this time. End the activity with encouragement to complete this activity on their own and post in a prominent location.

**Additional tips for maximal effectiveness:**

**Online interactive workshops with breakout rooms:**

- We recommend having a facilitator for each breakout room, but with adequate instruction and access to materials, it is possible for small groups to meet and the main speaker to go between breakout rooms, with support staff remaining in main meeting for questions.
- If there is time for reflection/conversation about challenges the participants may be facing, facilitators should invite other participants to share, and they should be prepared to share one challenge they experienced and explain how they overcame it.

**Online interactive workshops with other interactive components (chat, polls) and individual reflection:**

- Ways to incorporate interactive components through chat during didactic sessions:
  - Invite participants to share a character strength with the group (Breakout #1).
  - Invite participants to share ideas or phrases about vision statement, some may share pithy, ironic, or otherwise engaging visions that help others get creative (Breakout #2).
  - Invite participants to share one professional goal to “manifest” thoughts into outcomes (Breakout #3).
  - Invite questions through chat throughout the session for speaker(s) to address.
- Ways to incorporate polling into didactic sessions:
  - Consider creating a poll identifying professional roles (either current or desired) and have participants select the one that most resonates with their professional selves. We suggest the following (feel free to add or edit, according to your audience/goals of the sessions):
    - Clinician
    - Educator
    - Scholar
    - Patient advocate
    - Mentor or coach
- While we recommend as much interaction as possible for this session, self-reflection is a critical component so it could easily be adapted by providing more time for individual reflection. In which case, breakouts could be shortened slightly. Recommended alternative timing if only using individual reflections in place of breakout sessions:
  - Activity #1: 2-3 min
  - Activity #2: 10 min
  - Activity #3: 5-7 min

Please feel free to contact us with feedback or to share anecdotes from your workshop – wishing you a very successful session!

Sincerely,

Ryan, Shannon, and Emily

rnelson2@bidmc.harvard.edu
