# Supplementary material for: Develop Your CORE2 for Career Flourishing: A Career Development Workshop for Hospitalists
Source: MedEdPORTAL. 2024 Mar 15;20:11387. doi: 10.15766/mep_2374-8265.11387 (PMC10940547; doi:10.15766/mep_2374-8265.11387)
Supplement: Supplementary file 1 — Modules 1-4.pptxCharacter Strengths and Virtues Handout.docxParticipant Worksheet.docxGraphic Template.pptxFacilitator Guide.docxPresurvey.docxPostsurvey.docx [file mep_2374-8265.11387-s001.zip › F. Presurvey.docx]

1. Please answer the following demographic question:

☐ Check if you are a medical student. What is your MS year? (ex: 3^rd^ year: MS-3): MS______

☐ Check If you are a resident/fellow. What is your PGY year? (ex: 2^nd^ year: PGY-2): PGY______

☐ Check if you are a PA/NP student. What is your PA/NP year? (ex: 1^st^ year PA: PA-1): ______

☐ Check If you are a PA/NP. In what year did you begin practice? ______

☐ Check If you are a MD/DO. In what year did you begin practice? ______

1. How familiar are you with your signature character strengths?

| Not at all  familiar | Slightly  familiar | Moderately familiar | Very  familiar | Extremely  familiar |
| --- | --- | --- | --- | --- |
| 1 | 2 | 3 | 4 | 5 |

1. How would you rate the importance of writing a professional vision statement to your career development?

| Not at all  important | Slightly  important | Moderately  important | Very  important | Extremely  important |
| --- | --- | --- | --- | --- |
| 1 | 2 | 3 | 4 | 5 |

1. How would you rate your confidence in your ability to write a professional vision statement?

| Not at all confident | Slightly  confident | Moderately  confident | Very  confident | Extremely confident |
| --- | --- | --- | --- | --- |
| 1 | 2 | 3 | 4 | 5 |

1. How would you describe your knowledge of evidence-based principles to develop a SMART career goal?

| None | Minimal knowledge | Good  knowledge | Very good knowledge | Excellent knowledge |
| --- | --- | --- | --- | --- |
| 1 | 2 | 3 | 4 | 5 |

1. How would you rate your confidence in your ability to write a SMART career goal?

| Not at all confident | Slightly  confident | Moderately  confident | Very  confident | Extremely confident |
| --- | --- | --- | --- | --- |
| 1 | 2 | 3 | 4 | 5 |
