# Supplementary material for: Develop Your CORE2 for Career Flourishing: A Career Development Workshop for Hospitalists
Source: MedEdPORTAL. 2024 Mar 15;20:11387. doi: 10.15766/mep_2374-8265.11387 (PMC10940547; doi:10.15766/mep_2374-8265.11387)
Supplement: Supplementary file 1 — Modules 1-4.pptxCharacter Strengths and Virtues Handout.docxParticipant Worksheet.docxGraphic Template.pptxFacilitator Guide.docxPresurvey.docxPostsurvey.docx [file mep_2374-8265.11387-s001.zip › A. Modules 1-4.pptx]

## Slide 1
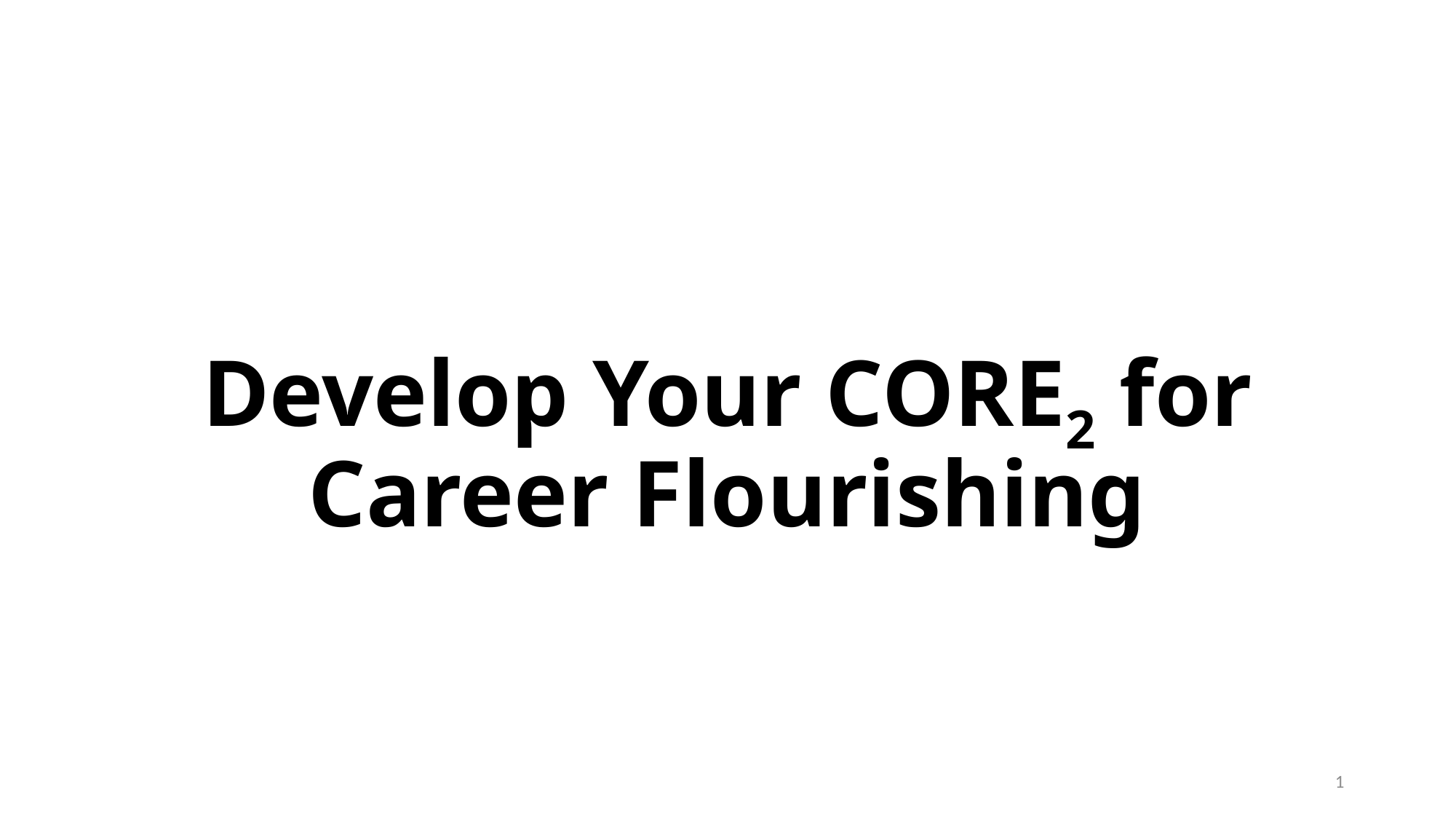

# Develop Your CORE2 for Career Flourishing
1

## Slide 2
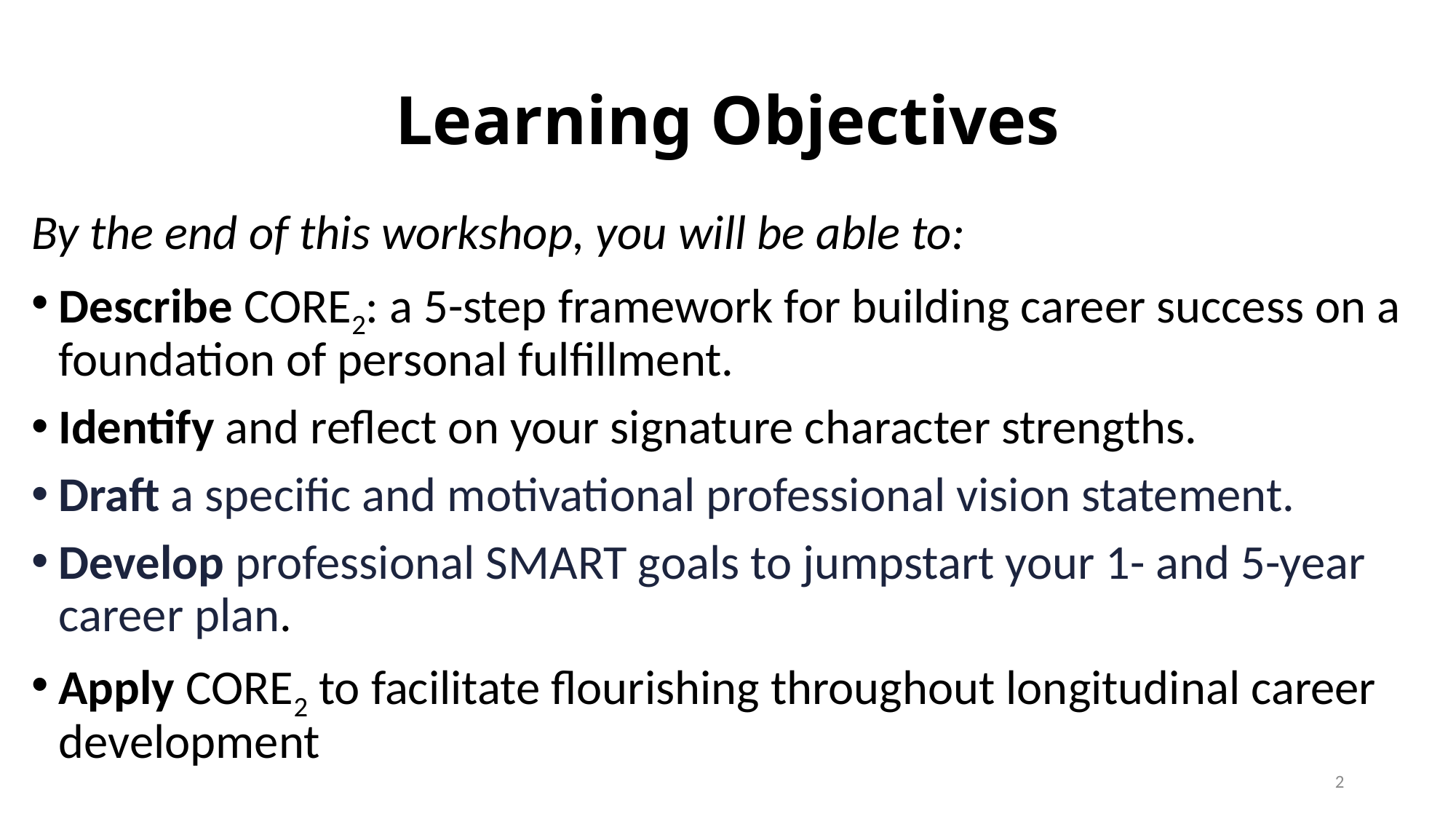

# Learning Objectives
By the end of this workshop, you will be able to:
Describe CORE2: a 5-step framework for building career success on a foundation of personal fulfillment.
Identify and reflect on your signature character strengths.
Draft a specific and motivational professional vision statement.
Develop professional SMART goals to jumpstart your 1- and 5-year career plan.
Apply CORE2 to facilitate flourishing throughout longitudinal career development
2

## Slide 3
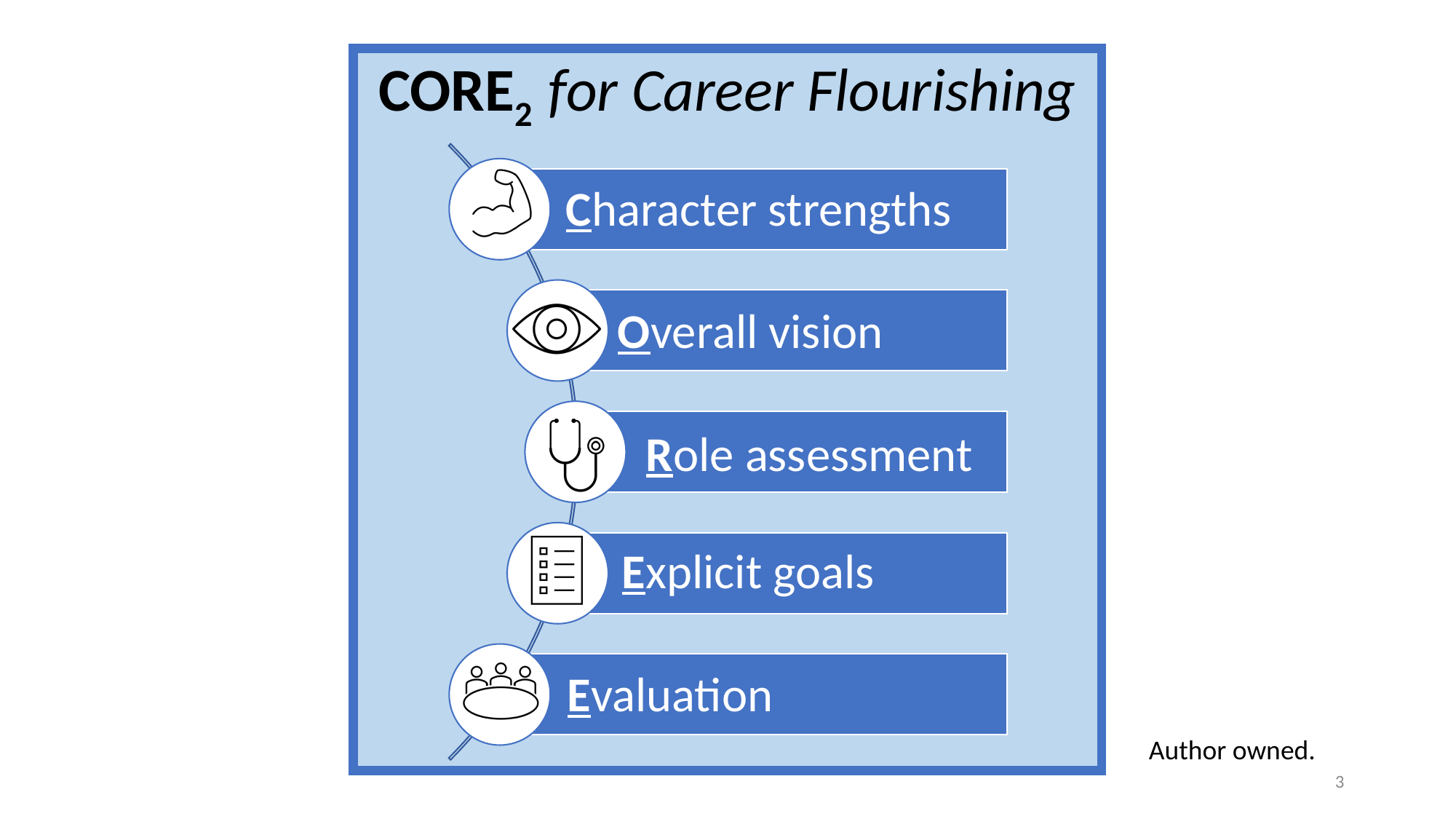

CORE2 for Career Flourishing
Character strengths
Overall vision
Role assessment
Explicit goals
Evaluation
Author owned.
3

## Slide 4
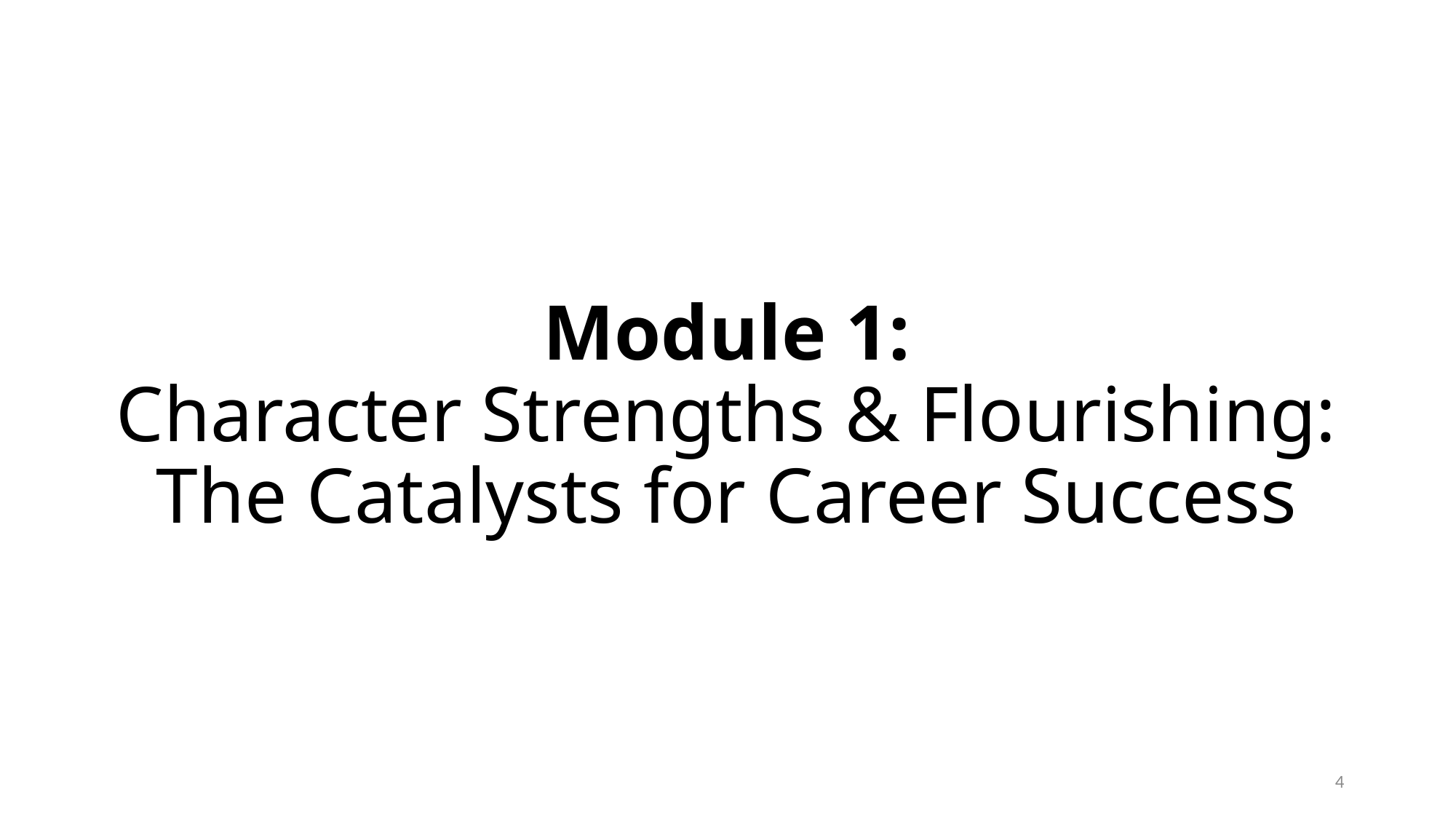

# Module 1: Character Strengths & Flourishing: The Catalysts for Career Success
4

## Slide 5
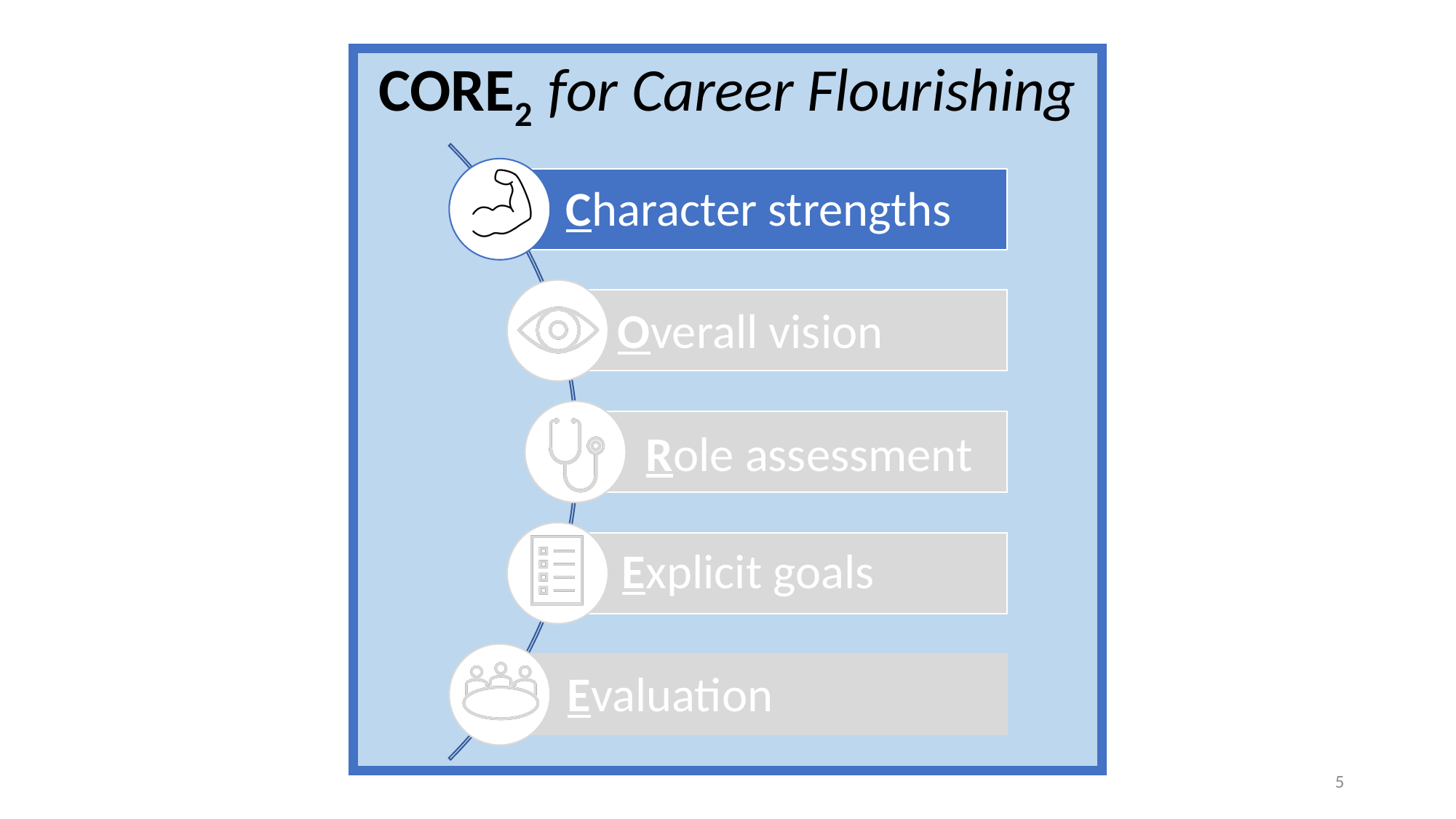

CORE2 for Career Flourishing
Character strengths
Overall vision
Role assessment
Explicit goals
Evaluation
5

## Slide 6
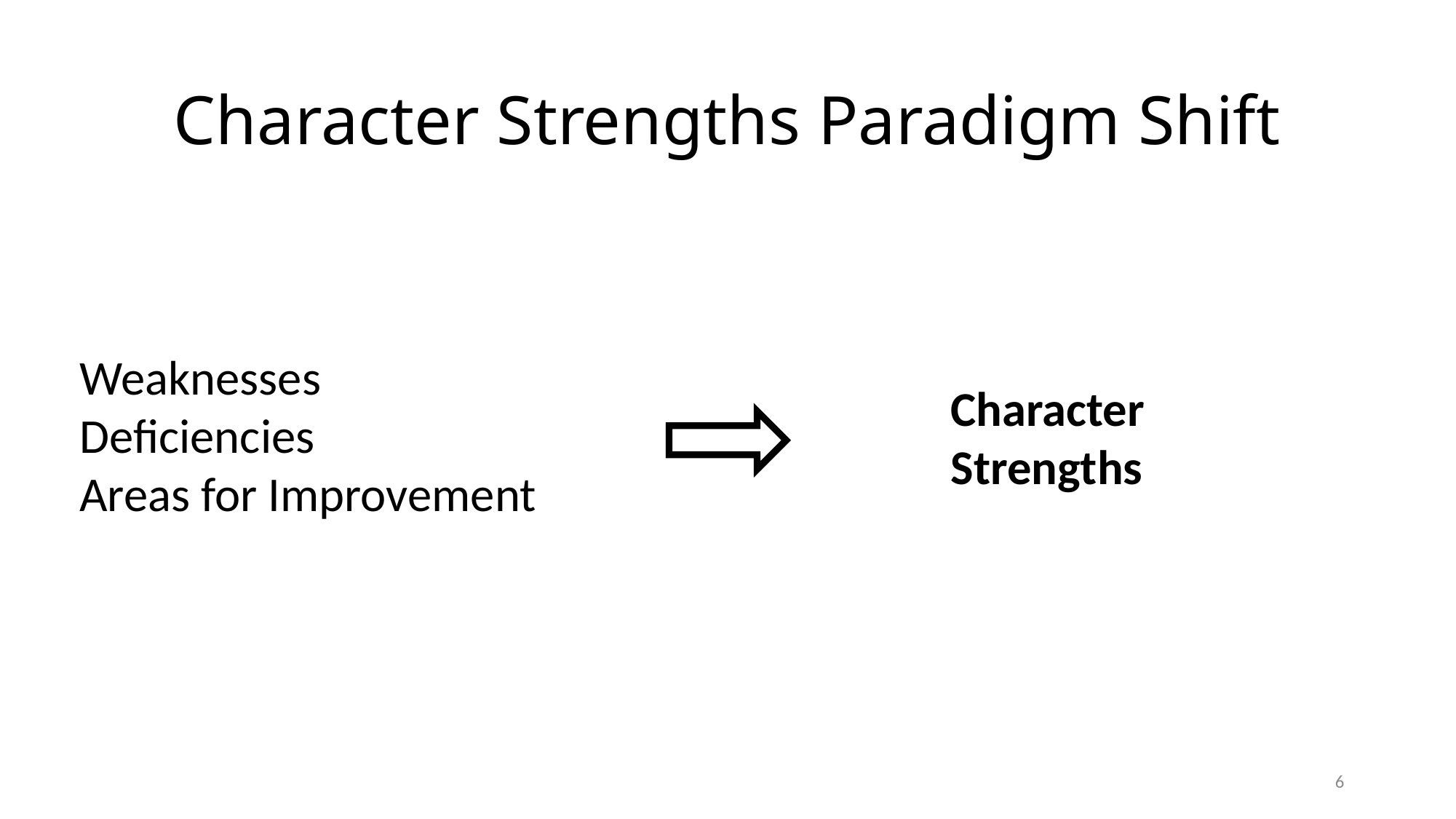

# Character Strengths Paradigm Shift
Weaknesses
Deficiencies
Areas for Improvement
Character Strengths
6

## Slide 7
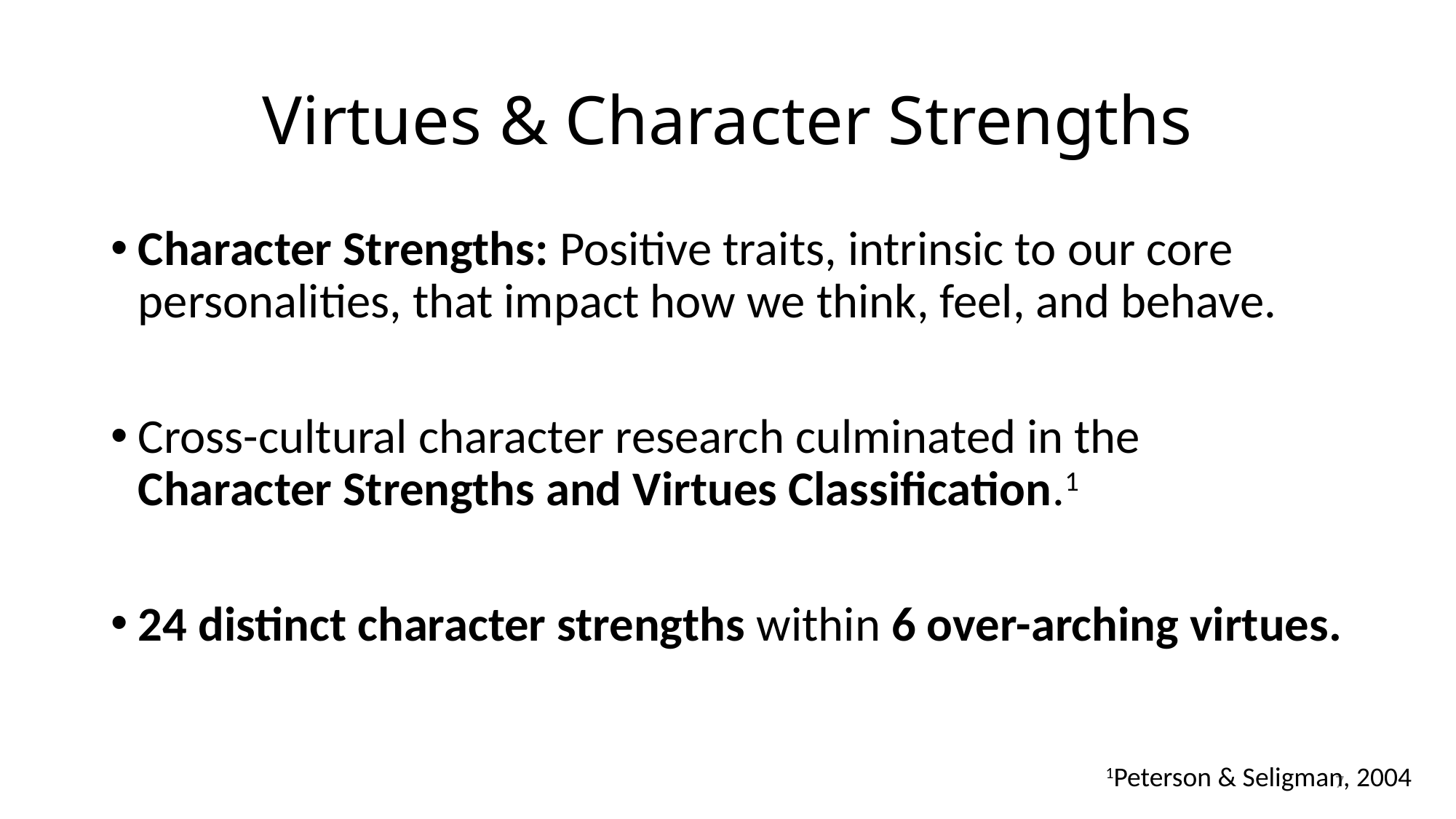

# Virtues & Character Strengths
Character Strengths: Positive traits, intrinsic to our core personalities, that impact how we think, feel, and behave.
Cross-cultural character research culminated in the Character Strengths and Virtues Classification.1
24 distinct character strengths within 6 over-arching virtues.
1Peterson & Seligman, 2004
7

## Slide 8
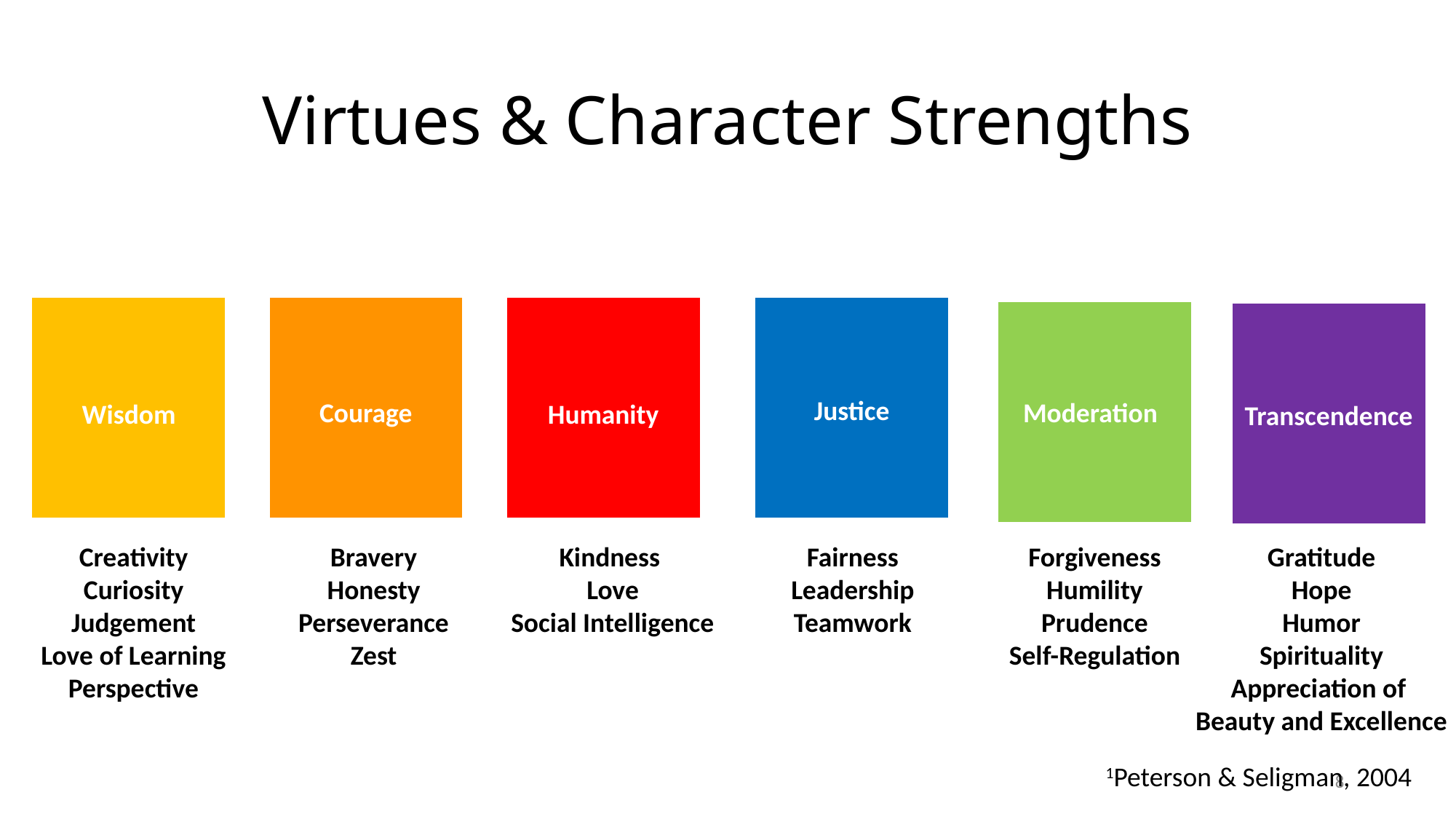

# Virtues & Character Strengths
Justice
Courage
Moderation
Wisdom
Humanity
Transcendence
Kindness
Love
Social Intelligence
Fairness
Leadership
Teamwork
Forgiveness
Humility
Prudence
Self-Regulation
Gratitude
Hope
Humor
Spirituality
Appreciation of
Beauty and Excellence
Bravery
Honesty
Perseverance
Zest
Creativity
Curiosity
Judgement
Love of Learning
Perspective
1Peterson & Seligman, 2004
8

## Slide 9
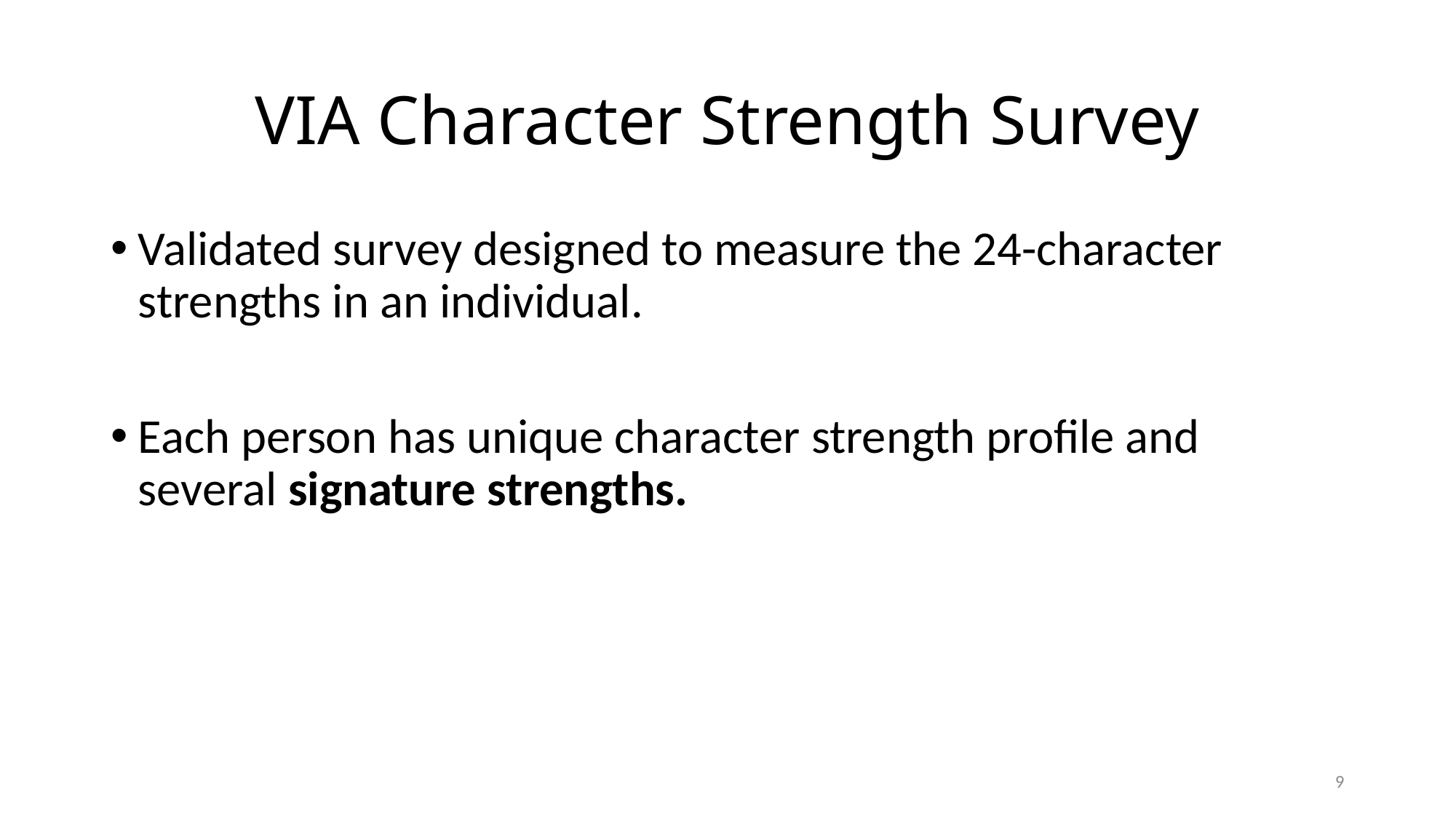

# VIA Character Strength Survey
Validated survey designed to measure the 24-character strengths in an individual.
Each person has unique character strength profile and several signature strengths.
9

## Slide 10
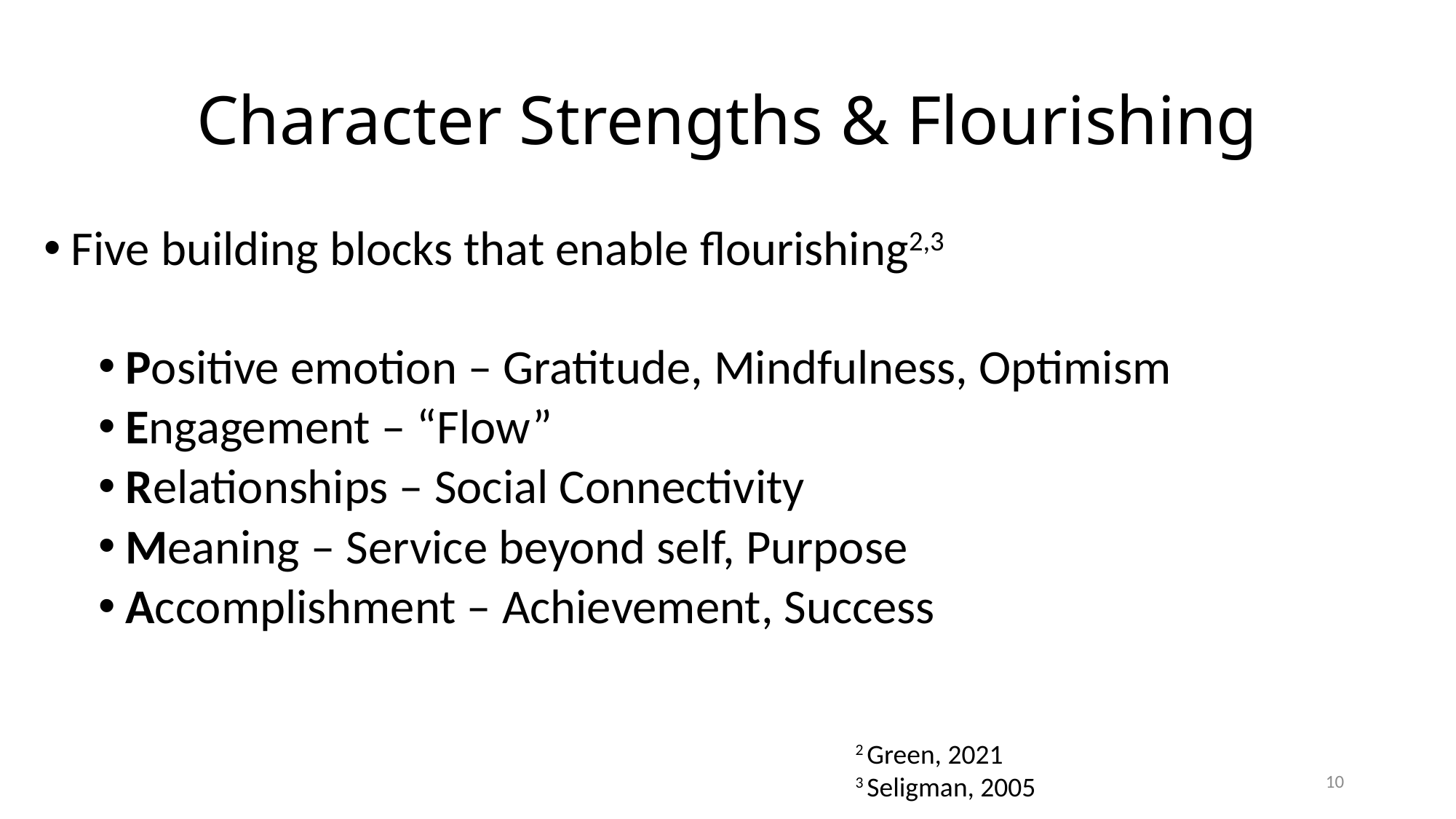

# Character Strengths & Flourishing
Five building blocks that enable flourishing2,3
Positive emotion – Gratitude, Mindfulness, Optimism
Engagement – “Flow”
Relationships – Social Connectivity
Meaning – Service beyond self, Purpose
Accomplishment – Achievement, Success
2 Green, 2021
3 Seligman, 2005
10

## Slide 11
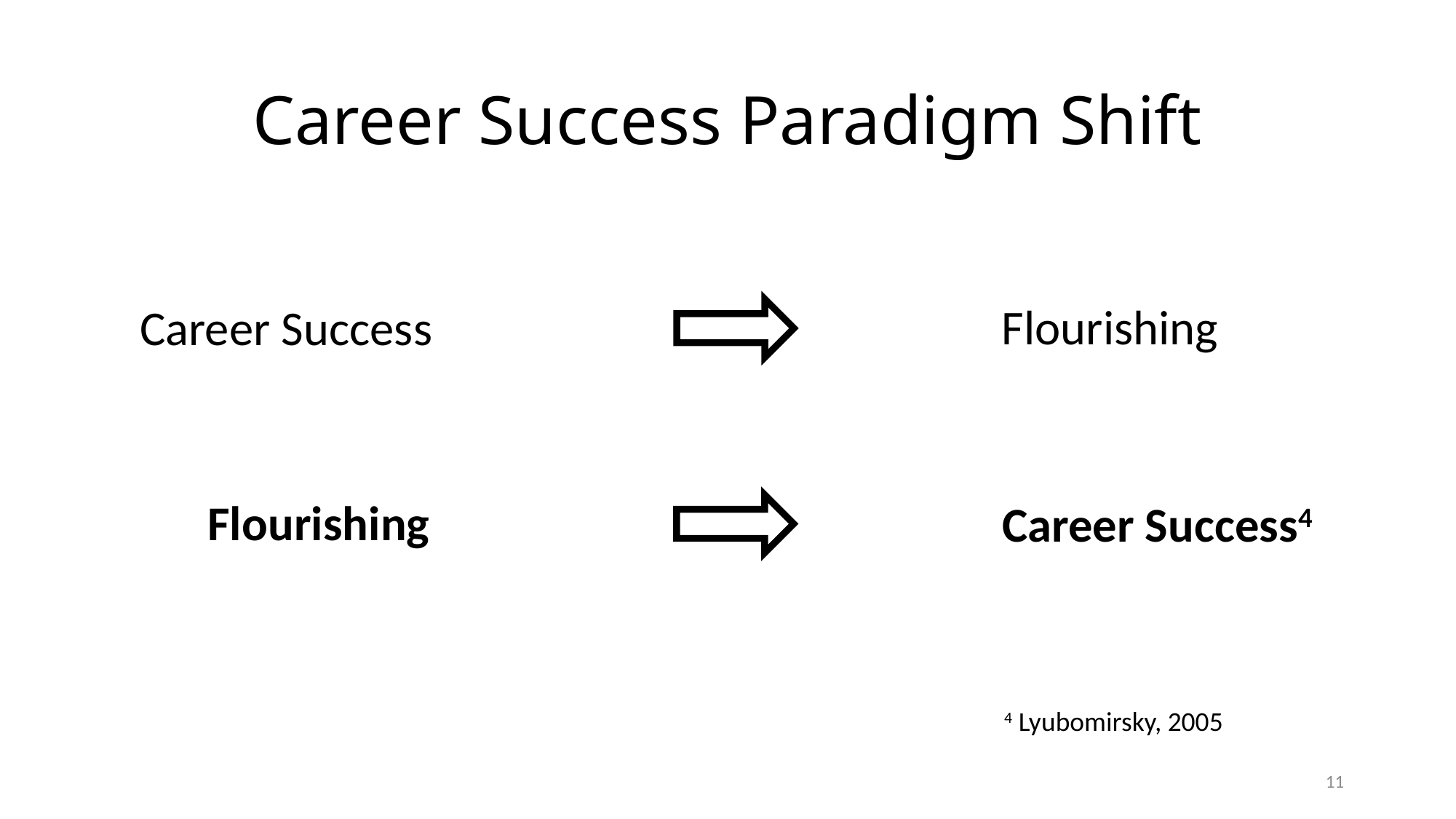

# Career Success Paradigm Shift
Flourishing
Career Success
Flourishing
Career Success4
 4 Lyubomirsky, 2005
11

## Slide 12
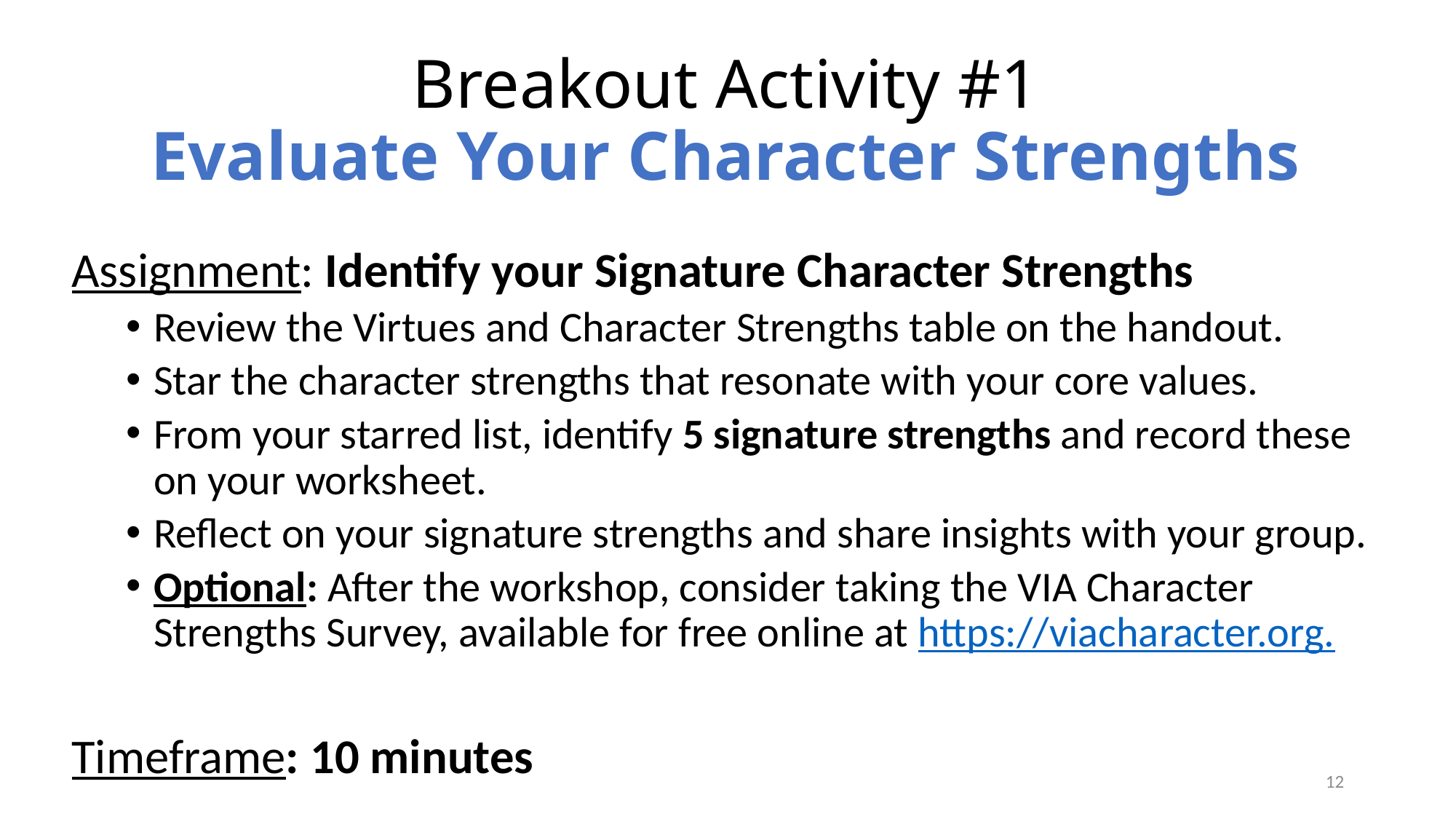

# Breakout Activity #1 Evaluate Your Character Strengths
Assignment: Identify your Signature Character Strengths
Review the Virtues and Character Strengths table on the handout.
Star the character strengths that resonate with your core values.
From your starred list, identify 5 signature strengths and record these on your worksheet.
Reflect on your signature strengths and share insights with your group.
Optional: After the workshop, consider taking the VIA Character Strengths Survey, available for free online at https://viacharacter.org.
Timeframe: 10 minutes
12

## Slide 13
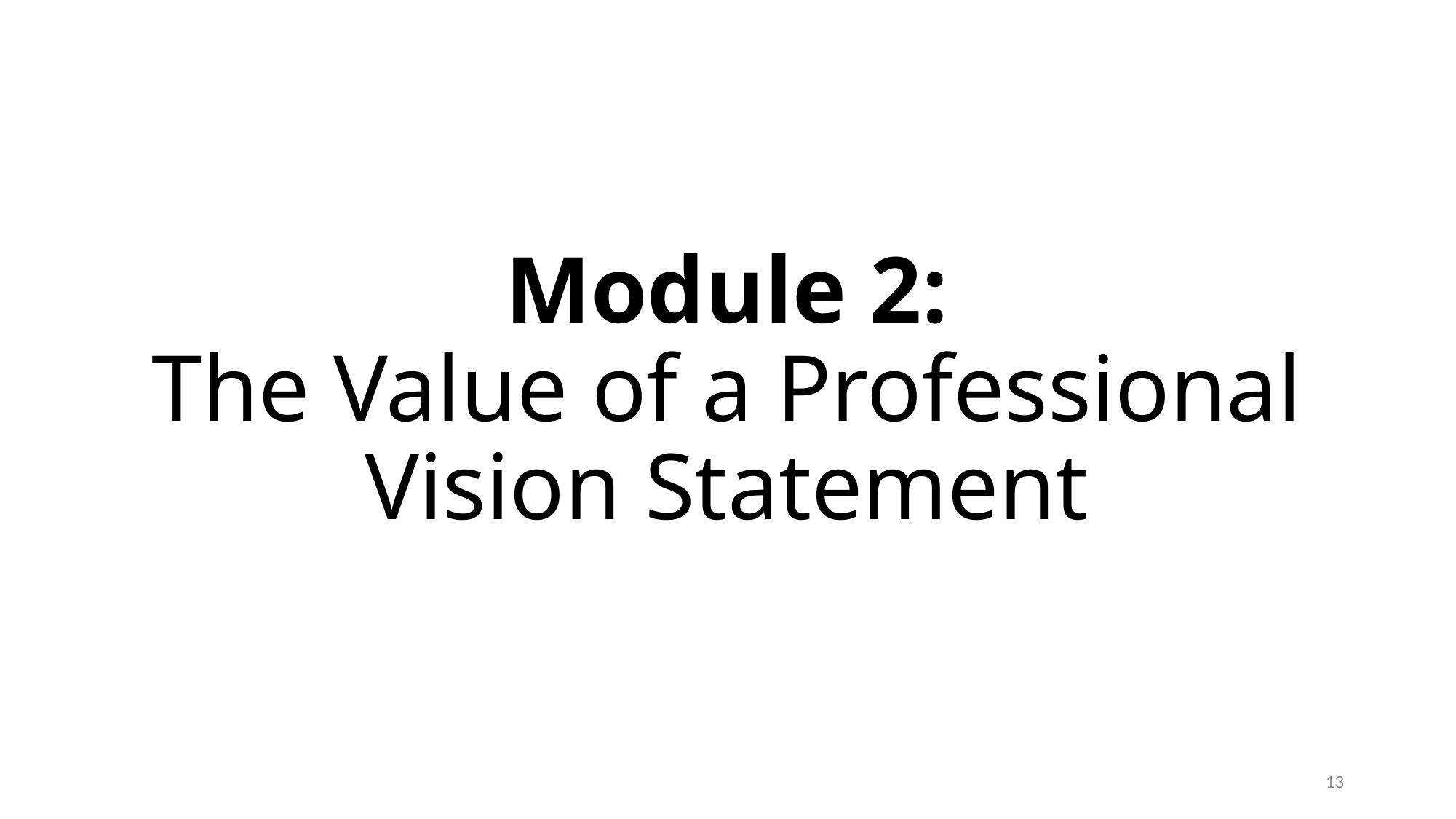

# Module 2: The Value of a Professional Vision Statement
13

## Slide 14
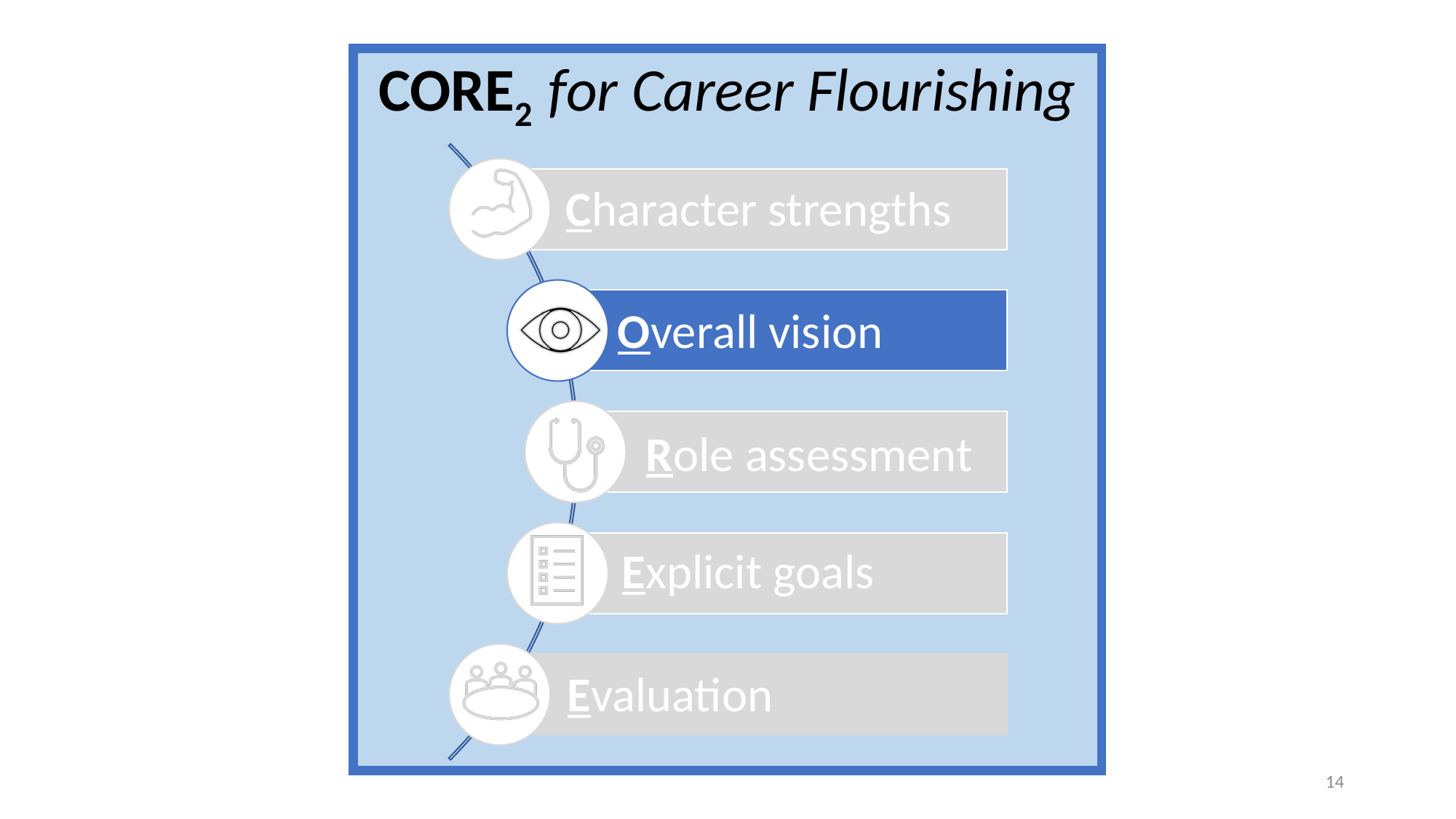

CORE2 for Career Flourishing
Character strengths
Overall vision
Role assessment
Explicit goals
Evaluation
14

## Slide 15
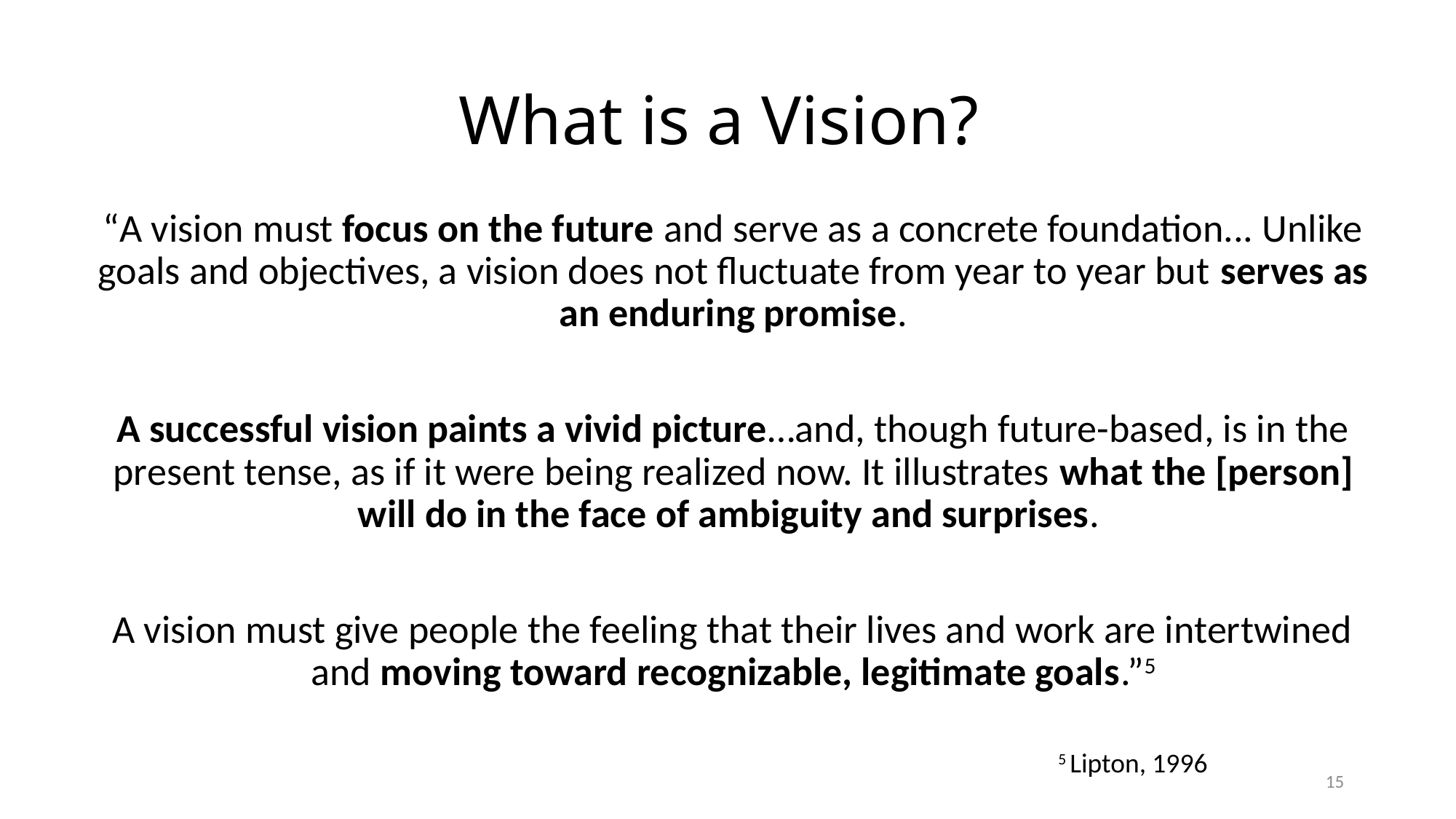

# What is a Vision?
“A vision must focus on the future and serve as a concrete foundation... Unlike goals and objectives, a vision does not fluctuate from year to year but serves as an enduring promise.
A successful vision paints a vivid picture…and, though future-based, is in the present tense, as if it were being realized now. It illustrates what the [person] will do in the face of ambiguity and surprises.
A vision must give people the feeling that their lives and work are intertwined and moving toward recognizable, legitimate goals.”5
5 Lipton, 1996
15

## Slide 16
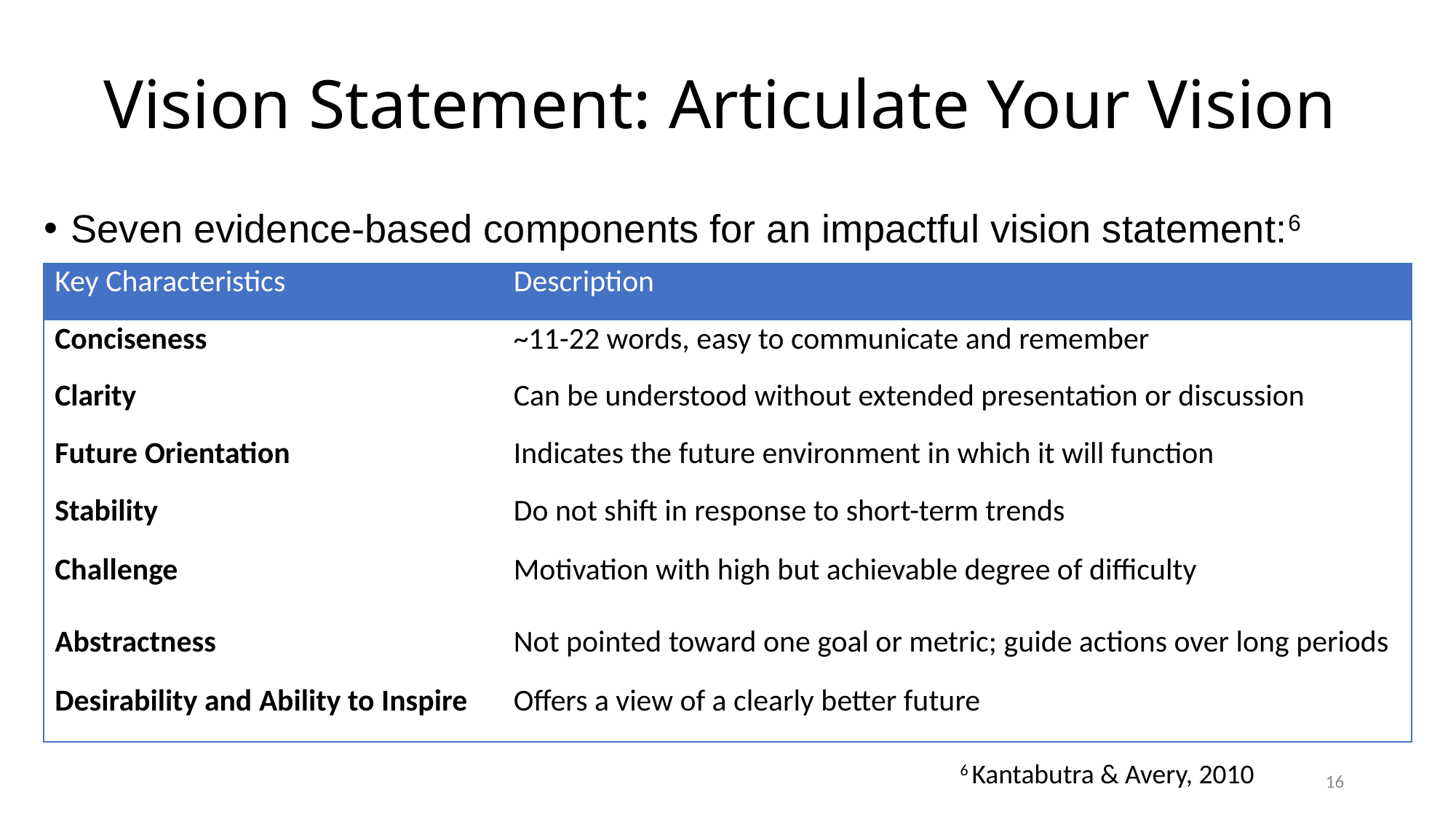

# Vision Statement: Articulate Your Vision
Seven evidence-based components for an impactful vision statement:6
| Key Characteristics | Description |
| --- | --- |
| Conciseness | ~11-22 words, easy to communicate and remember |
| Clarity | Can be understood without extended presentation or discussion |
| Future Orientation | Indicates the future environment in which it will function |
| Stability | Do not shift in response to short-term trends |
| Challenge | Motivation with high but achievable degree of difficulty |
| Abstractness | Not pointed toward one goal or metric; guide actions over long periods |
| Desirability and Ability to Inspire | Offers a view of a clearly better future |
6 Kantabutra & Avery, 2010
16

## Slide 17
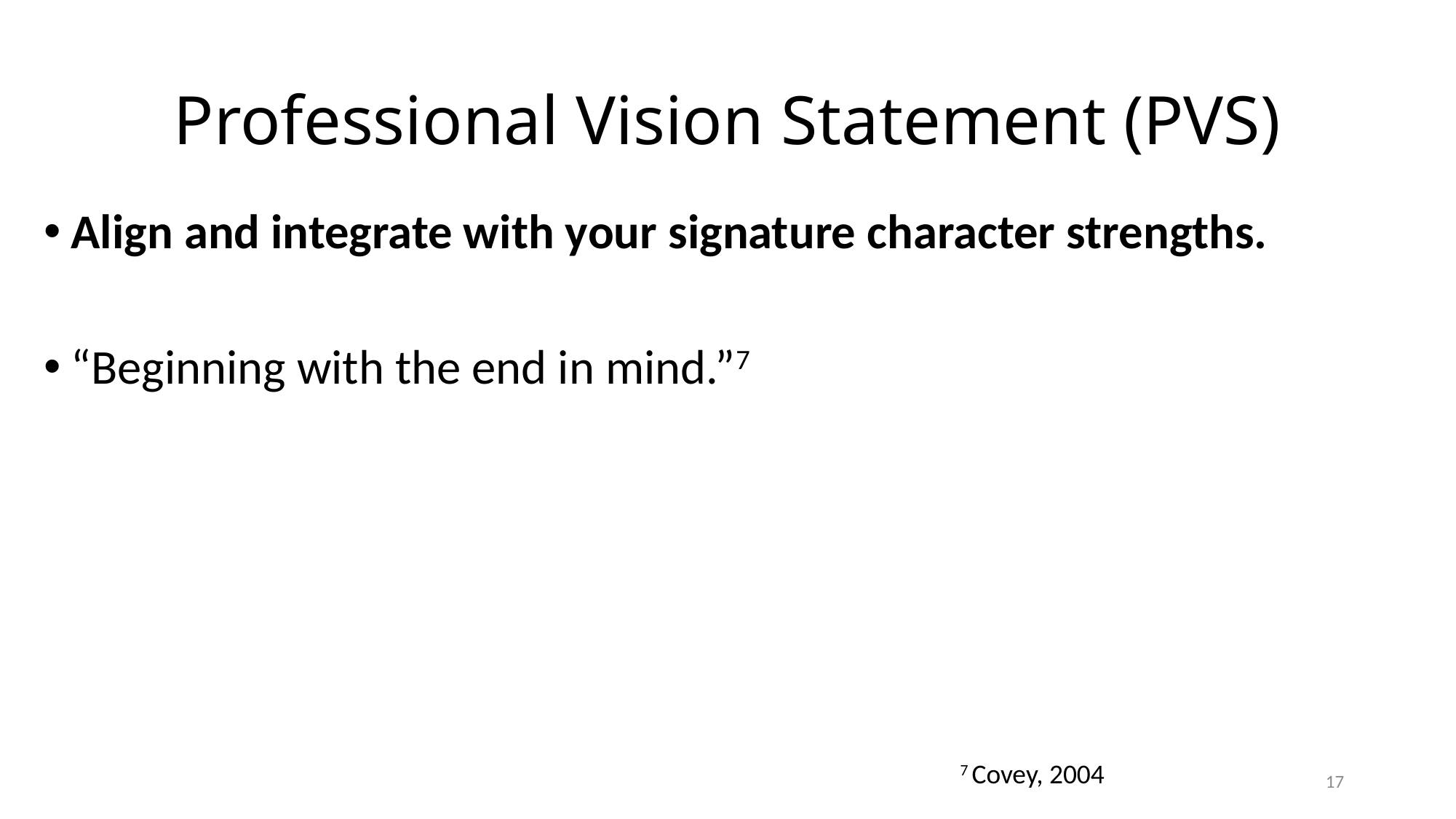

# Professional Vision Statement (PVS)
Align and integrate with your signature character strengths.
“Beginning with the end in mind.”7
7 Covey, 2004
17

## Slide 18
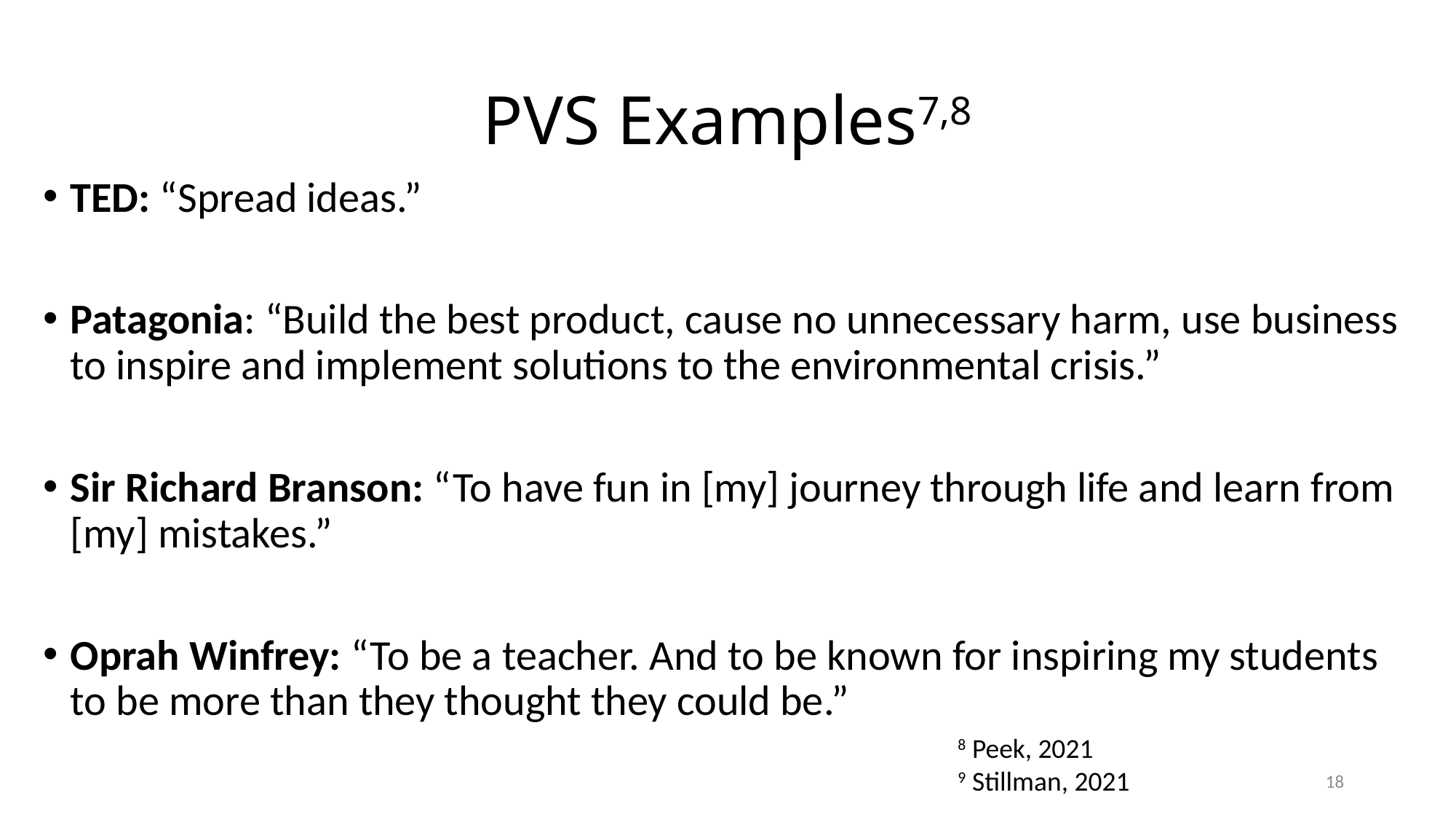

# PVS Examples7,8
TED: “Spread ideas.”
Patagonia: “Build the best product, cause no unnecessary harm, use business to inspire and implement solutions to the environmental crisis.”
Sir Richard Branson: “To have fun in [my] journey through life and learn from [my] mistakes.”
Oprah Winfrey: “To be a teacher. And to be known for inspiring my students to be more than they thought they could be.”
8 Peek, 2021
9 Stillman, 2021
18

## Slide 19
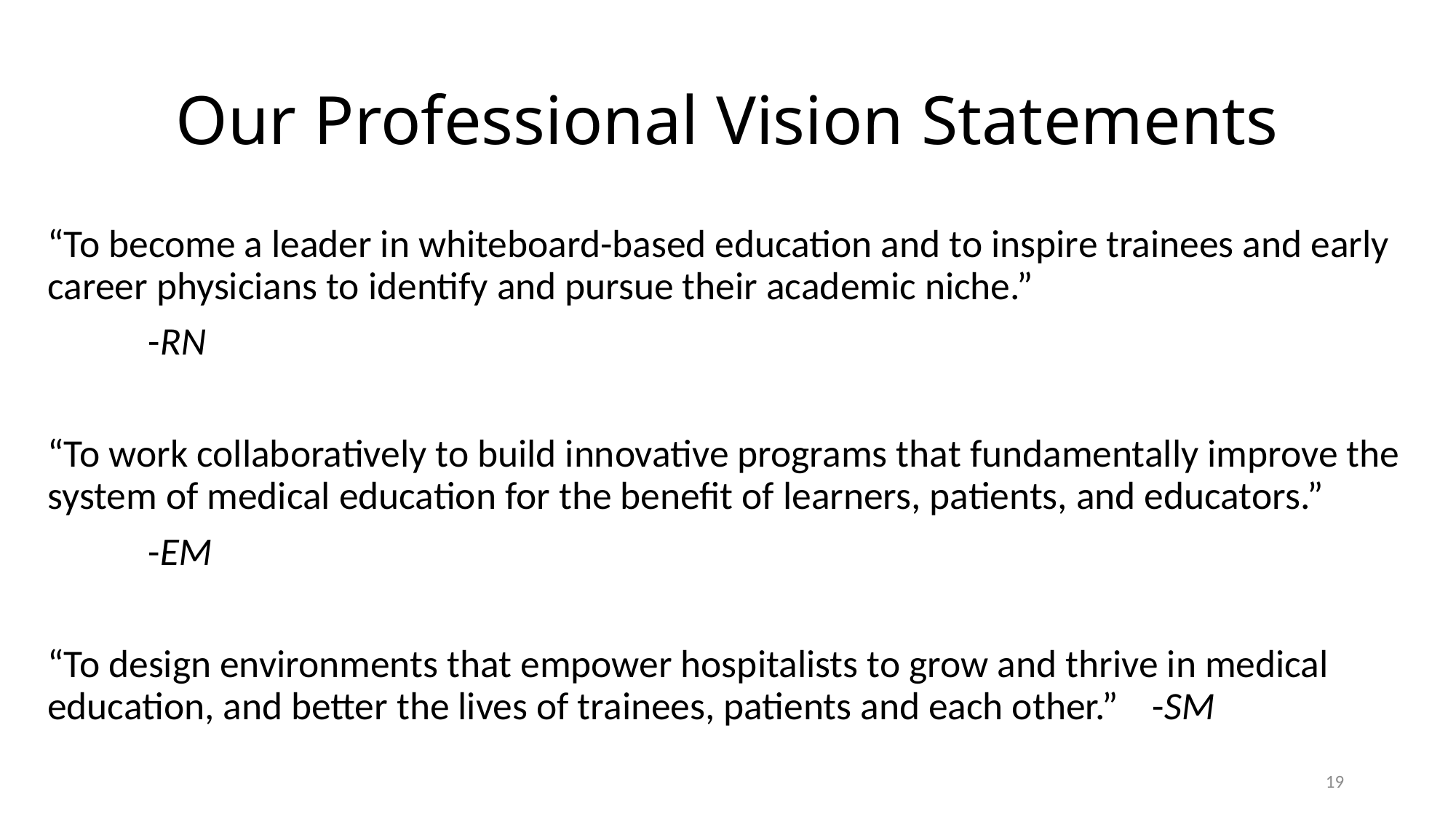

# Our Professional Vision Statements
“To become a leader in whiteboard-based education and to inspire trainees and early career physicians to identify and pursue their academic niche.”
											-RN
“To work collaboratively to build innovative programs that fundamentally improve the system of medical education for the benefit of learners, patients, and educators.”
											-EM
“To design environments that empower hospitalists to grow and thrive in medical education, and better the lives of trainees, patients and each other.” 													-SM
19

## Slide 20
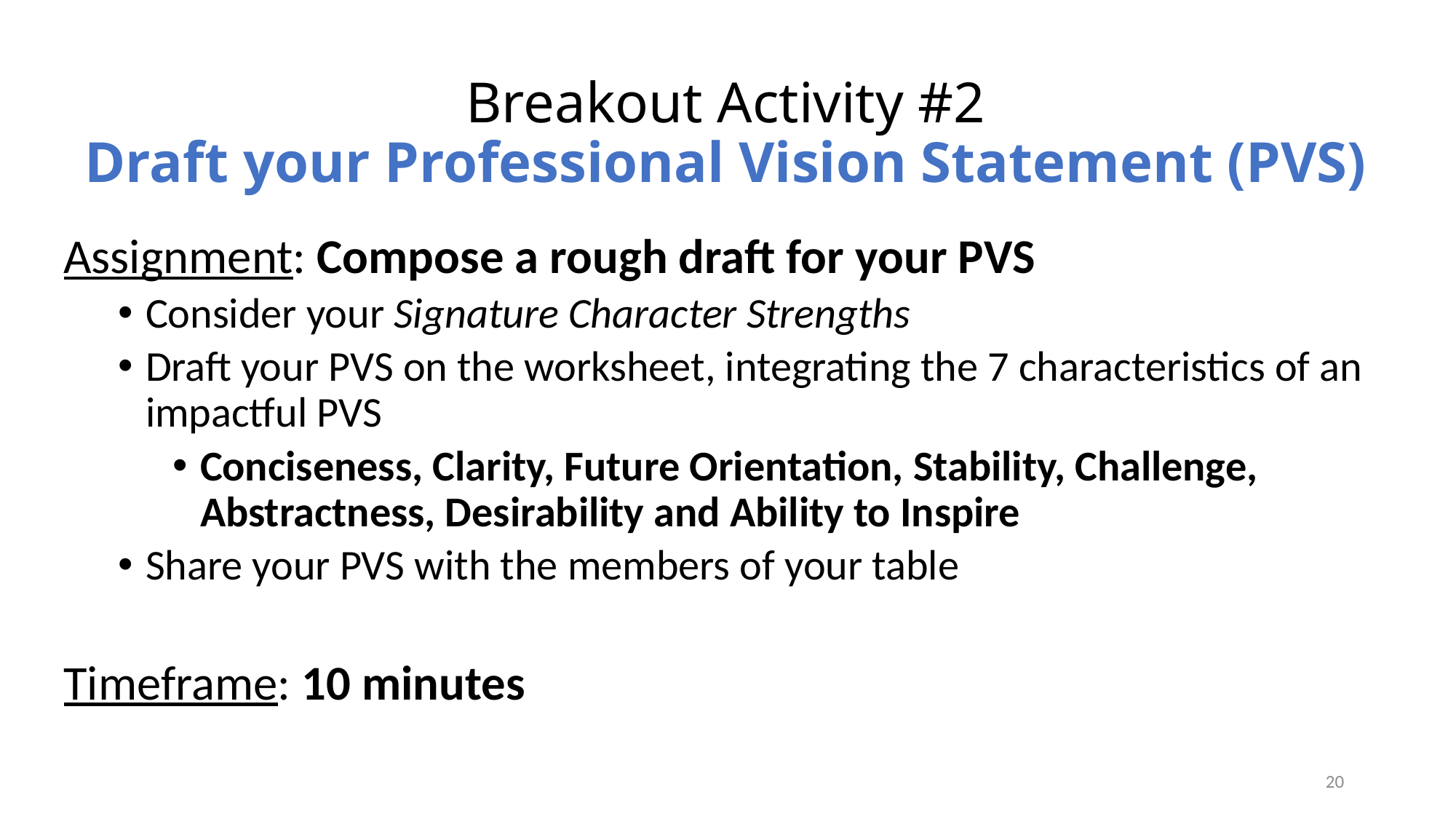

# Breakout Activity #2 Draft your Professional Vision Statement (PVS)
Assignment: Compose a rough draft for your PVS
Consider your Signature Character Strengths
Draft your PVS on the worksheet, integrating the 7 characteristics of an impactful PVS
Conciseness, Clarity, Future Orientation, Stability, Challenge, Abstractness, Desirability and Ability to Inspire
Share your PVS with the members of your table
Timeframe: 10 minutes
20

## Slide 21
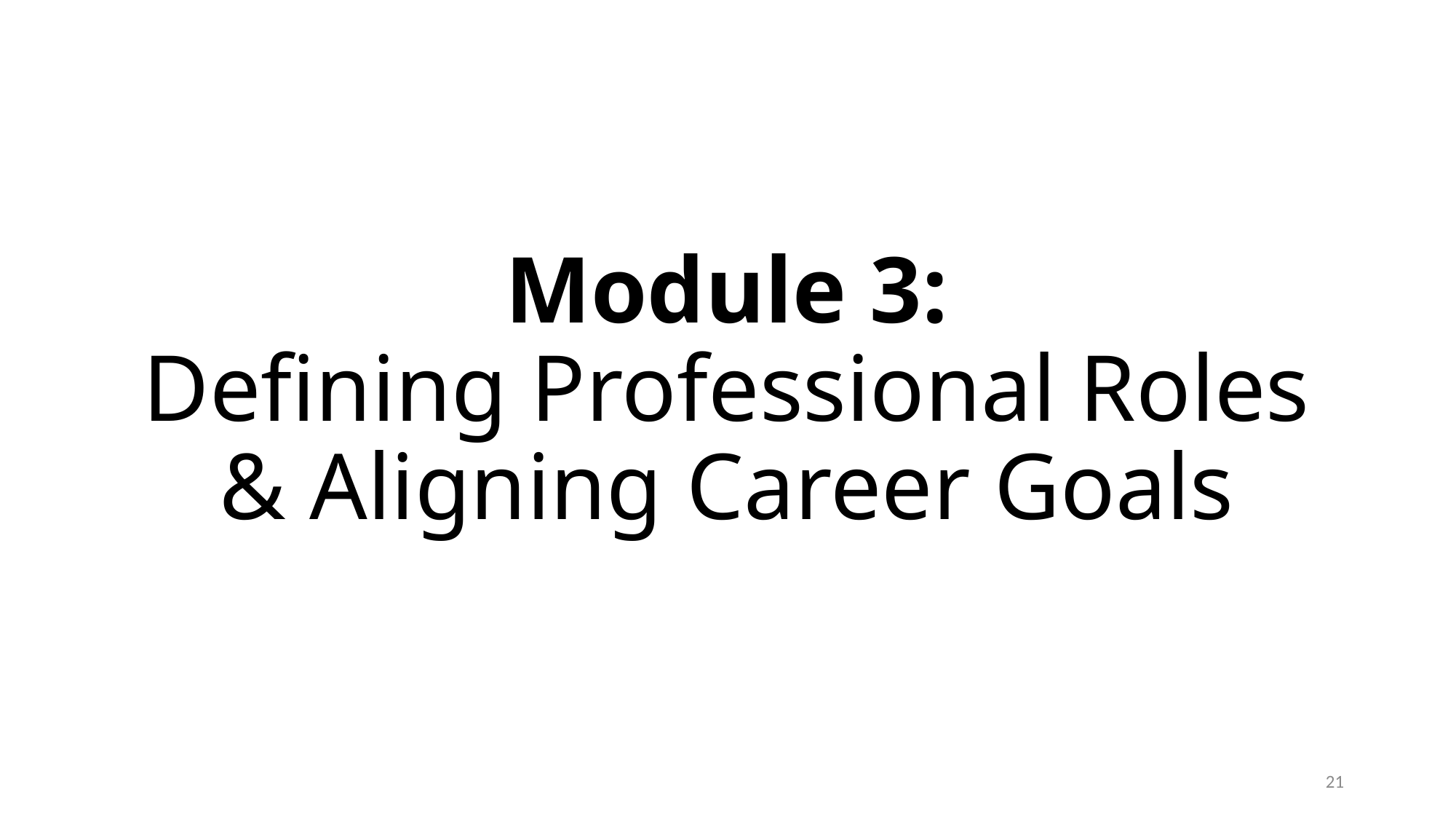

# Module 3: Defining Professional Roles & Aligning Career Goals
21

## Slide 22
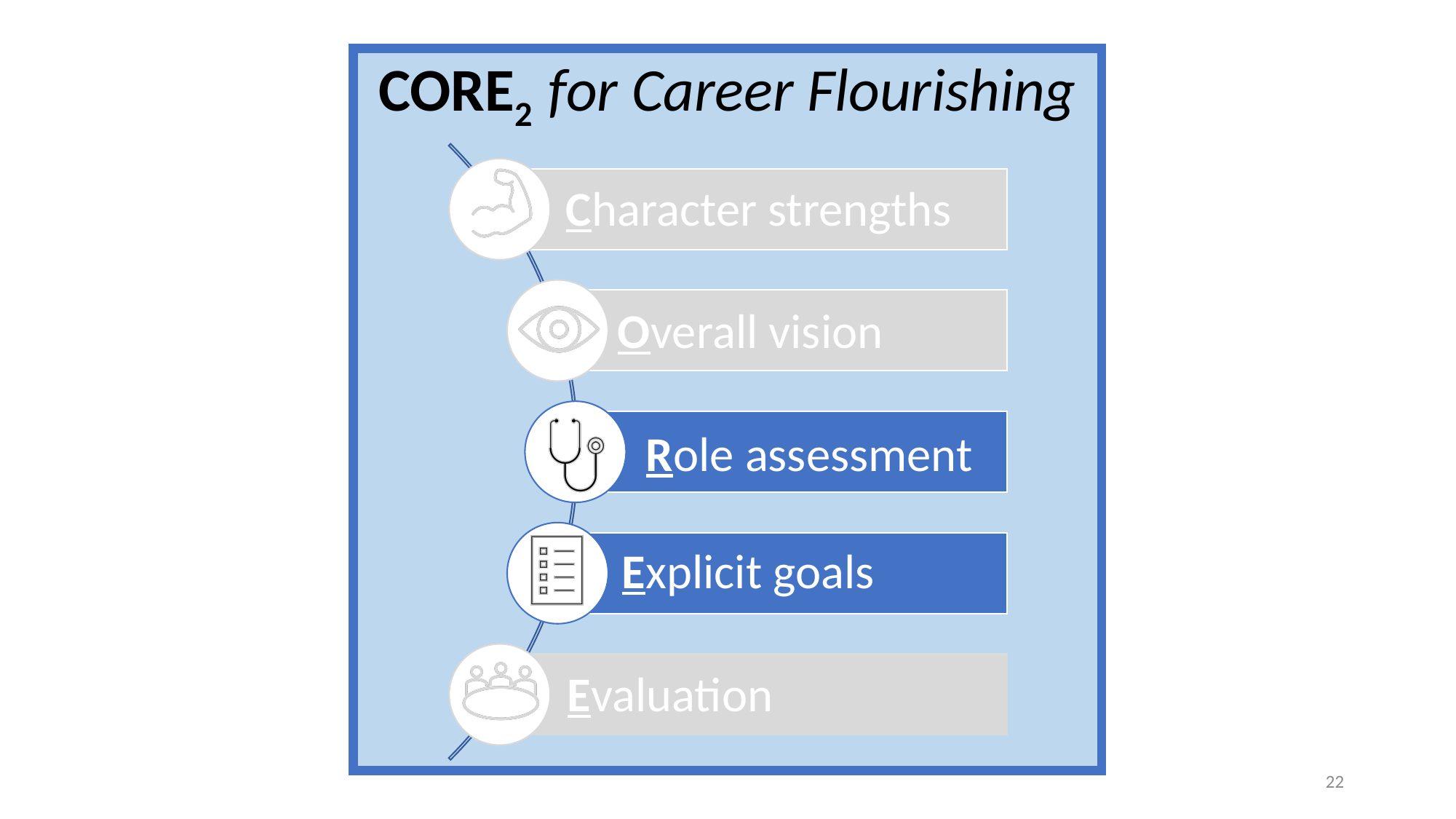

CORE2 for Career Flourishing
Character strengths
Overall vision
Role assessment
Explicit goals
Evaluation
22

## Slide 23
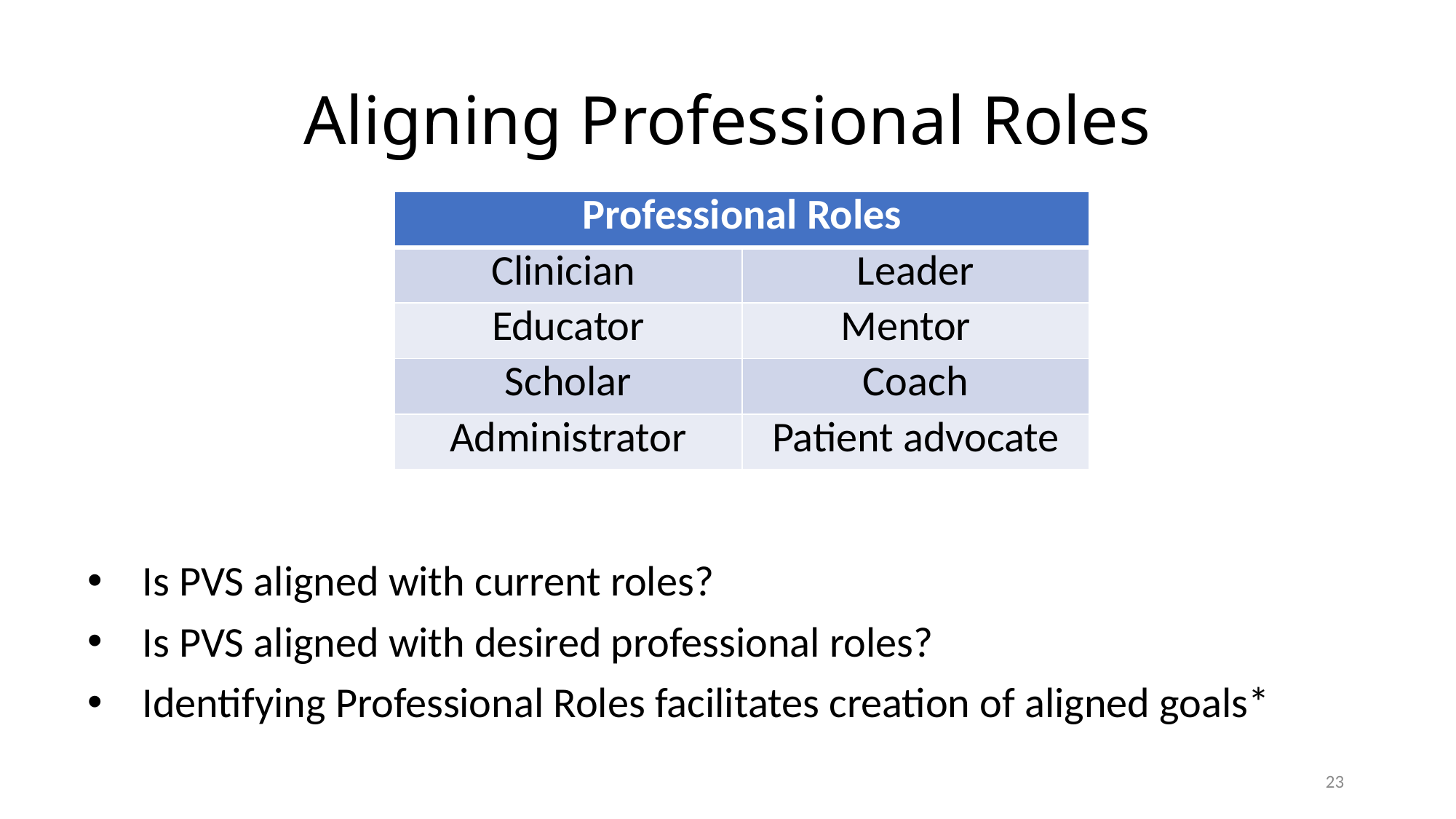

# Aligning Professional Roles
| Professional Roles | |
| --- | --- |
| Clinician | Leader |
| Educator | Mentor |
| Scholar | Coach |
| Administrator | Patient advocate |
Is PVS aligned with current roles?
Is PVS aligned with desired professional roles?
Identifying Professional Roles facilitates creation of aligned goals*
23

## Slide 24
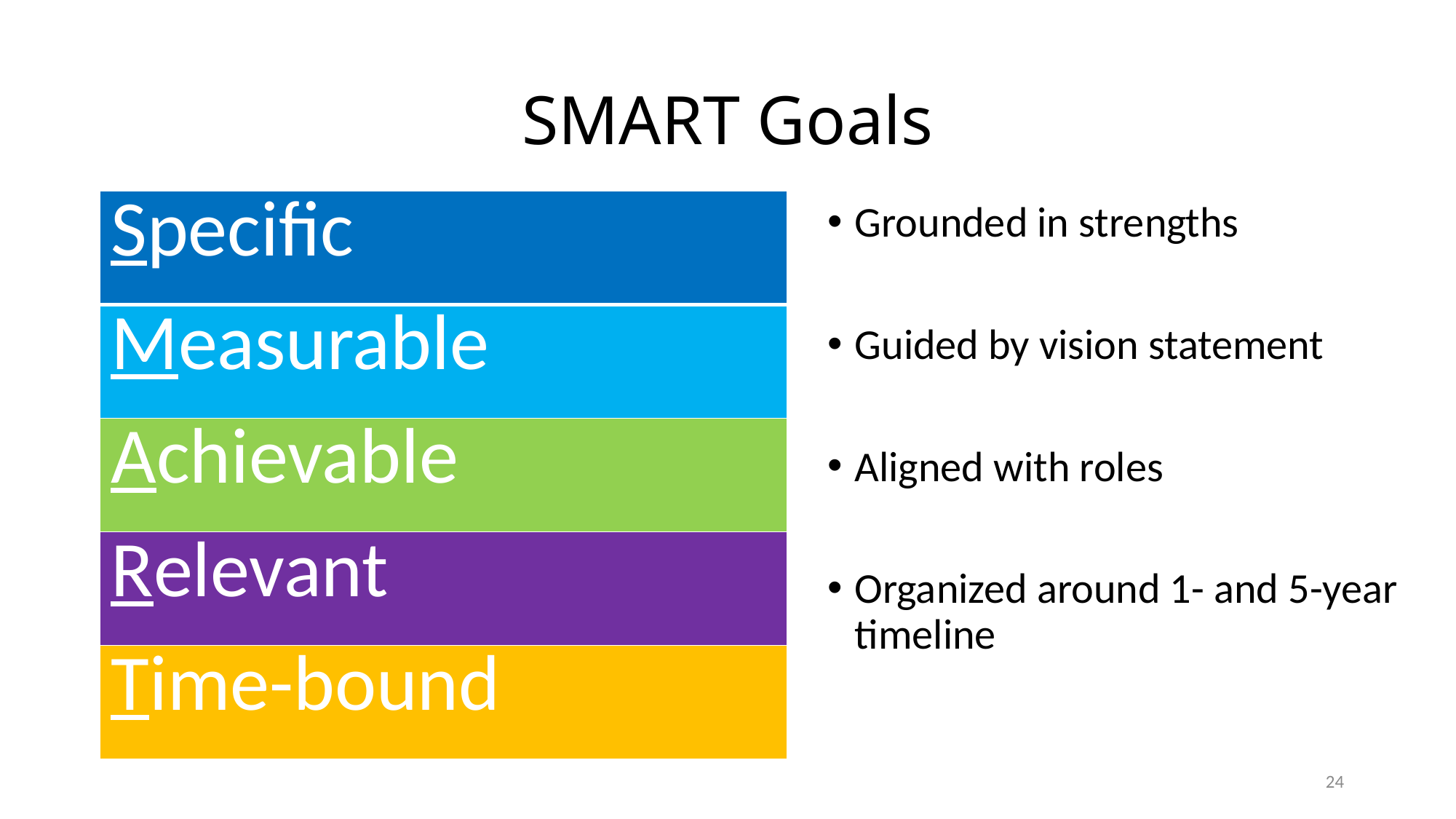

# SMART Goals
| Specific |
| --- |
| Measurable |
| Achievable |
| Relevant |
| Time-bound |
Grounded in strengths
Guided by vision statement
Aligned with roles
Organized around 1- and 5-year timeline
24

## Slide 25
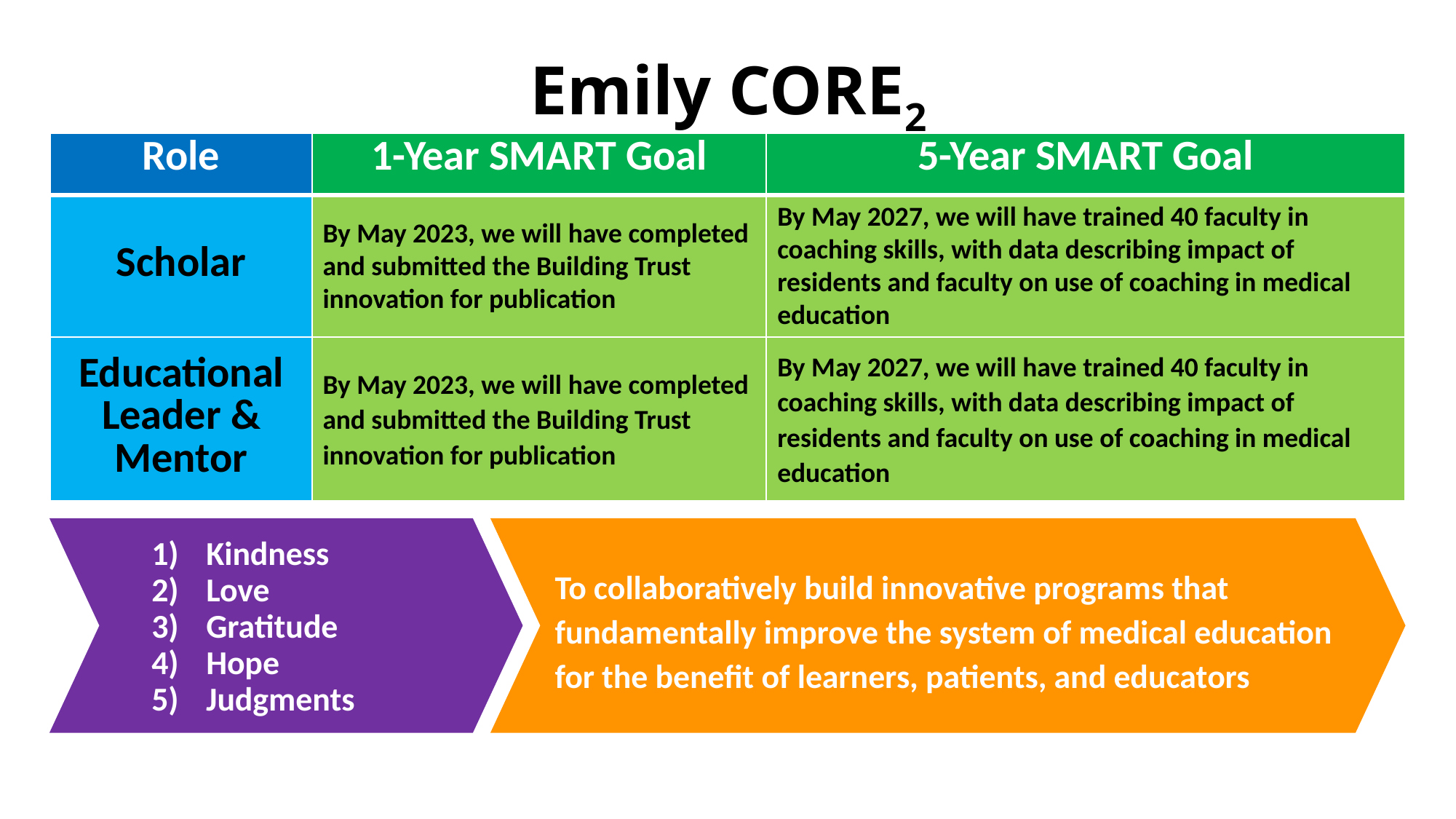

Emily CORE2
| Role | 1-Year SMART Goal | 5-Year SMART Goal |
| --- | --- | --- |
| Scholar | By May 2023, we will have completed and submitted the Building Trust innovation for publication​ | By May 2027, we will have trained 40 faculty in coaching skills, with data describing impact of residents and faculty on use of coaching in medical education |
| Educational Leader & Mentor | By May 2023, we will have completed and submitted the Building Trust innovation for publication | By May 2027, we will have trained 40 faculty in coaching skills, with data describing impact of residents and faculty on use of coaching in medical education |
Kindness
Love
Gratitude​
Hope
Judgments
To collaboratively build innovative programs that fundamentally improve the system of medical education for the benefit of learners, patients, and educators

## Slide 26
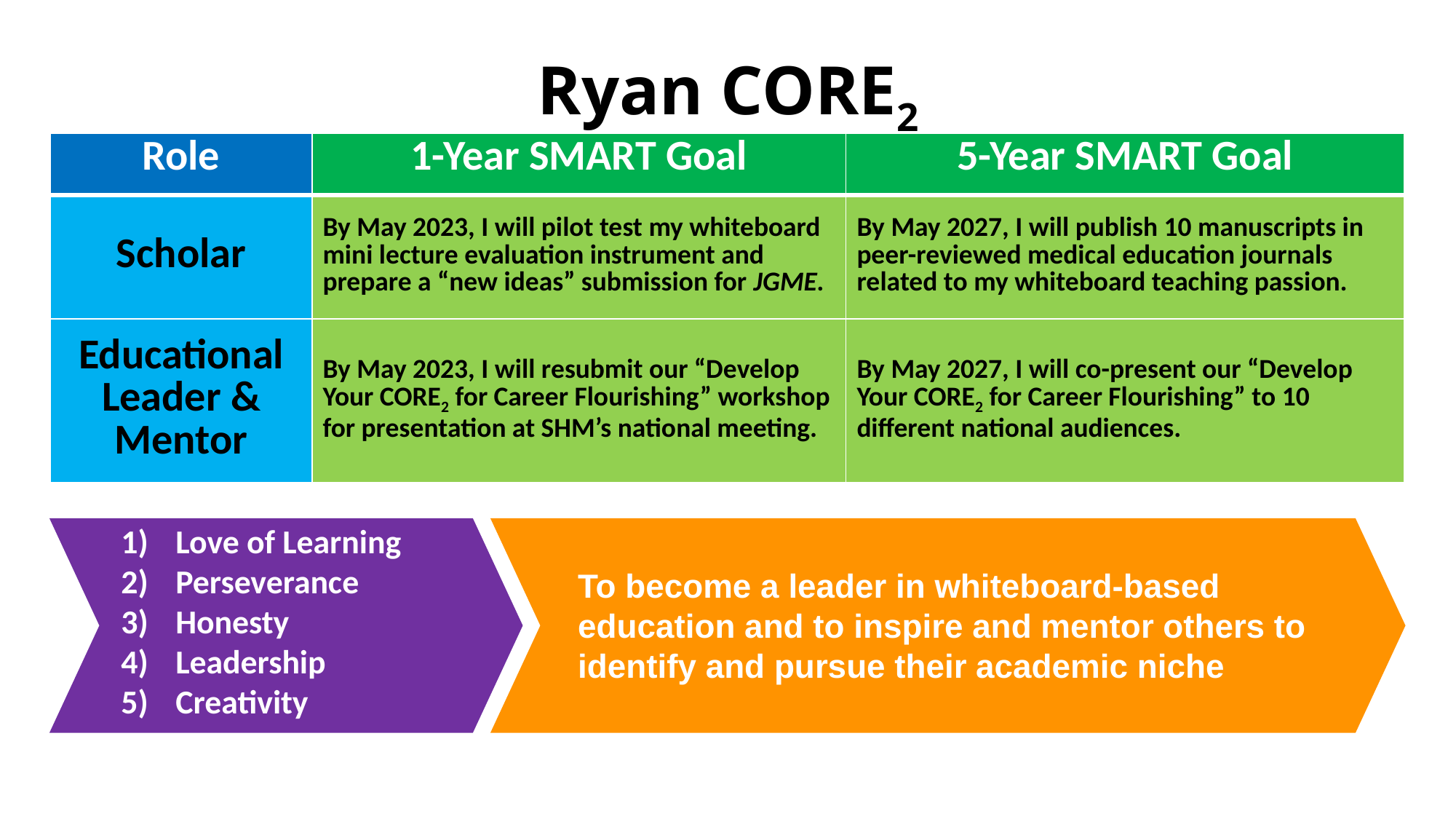

Ryan CORE2
| Role | 1-Year SMART Goal | 5-Year SMART Goal |
| --- | --- | --- |
| Scholar | By May 2023, I will pilot test my whiteboard mini lecture evaluation instrument and prepare a “new ideas” submission for JGME. | By May 2027, I will publish 10 manuscripts in peer-reviewed medical education journals related to my whiteboard teaching passion. |
| Educational Leader & Mentor | By May 2023, I will resubmit our “Develop Your CORE2 for Career Flourishing” workshop for presentation at SHM’s national meeting. | By May 2027, I will co-present our “Develop Your CORE2 for Career Flourishing” to 10 different national audiences. |
Love of Learning
Perseverance
Honesty
Leadership
Creativity
To become a leader in whiteboard-based education and to inspire and mentor others to identify and pursue their academic niche

## Slide 27
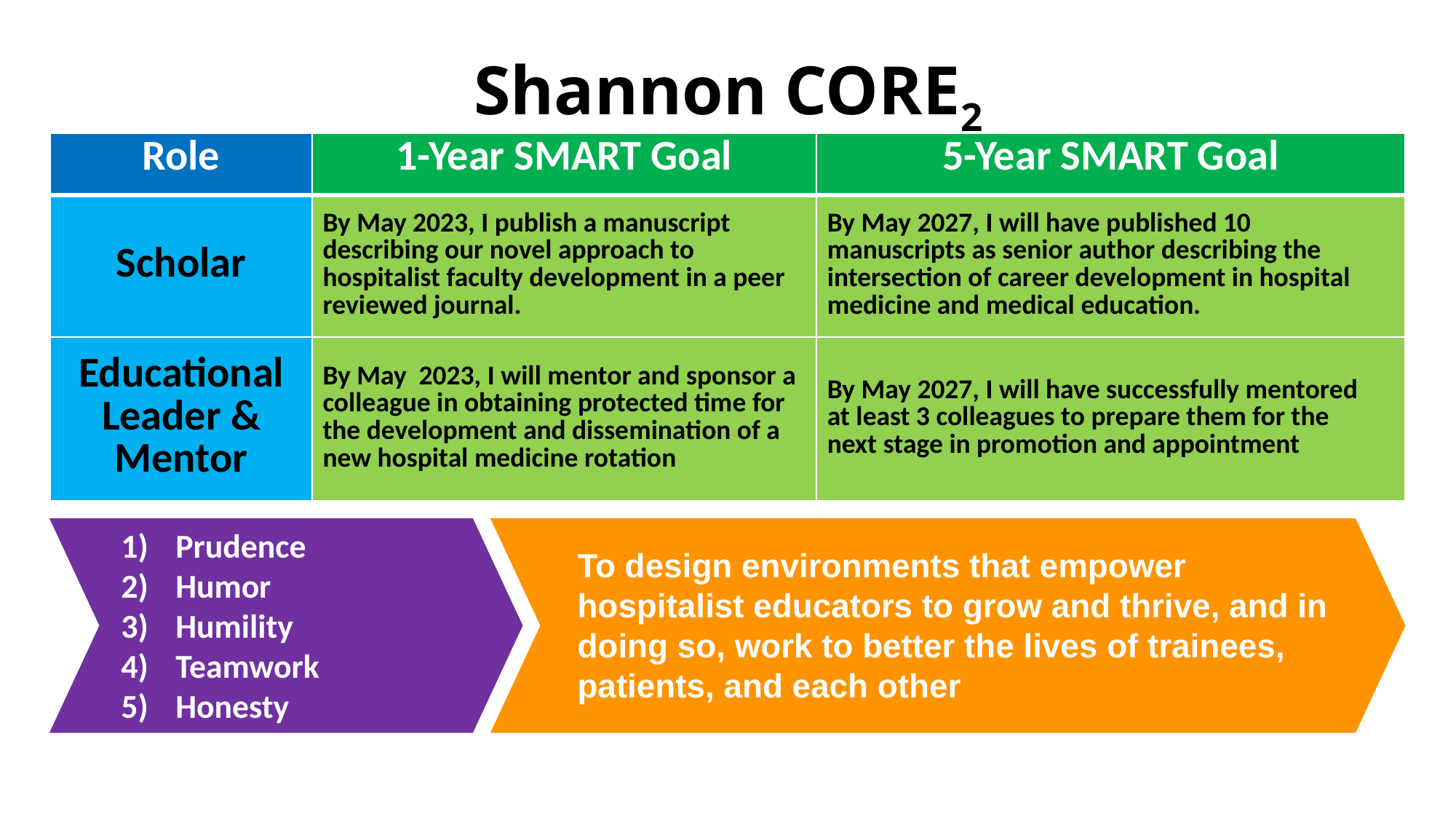

Shannon CORE2
| Role | 1-Year SMART Goal | 5-Year SMART Goal |
| --- | --- | --- |
| Scholar | By May 2023, I publish a manuscript describing our novel approach to hospitalist faculty development in a peer reviewed journal. | By May 2027, I will have published 10 manuscripts as senior author describing the intersection of career development in hospital medicine and medical education. |
| Educational Leader & Mentor | By May 2023, I will mentor and sponsor a colleague in obtaining protected time for the development and dissemination of a new hospital medicine rotation | By May 2027, I will have successfully mentored at least 3 colleagues to prepare them for the next stage in promotion and appointment |
Prudence
Humor
Humility
Teamwork
Honesty
To design environments that empower hospitalist educators to grow and thrive, and in doing so, work to better the lives of trainees, patients, and each other

## Slide 28
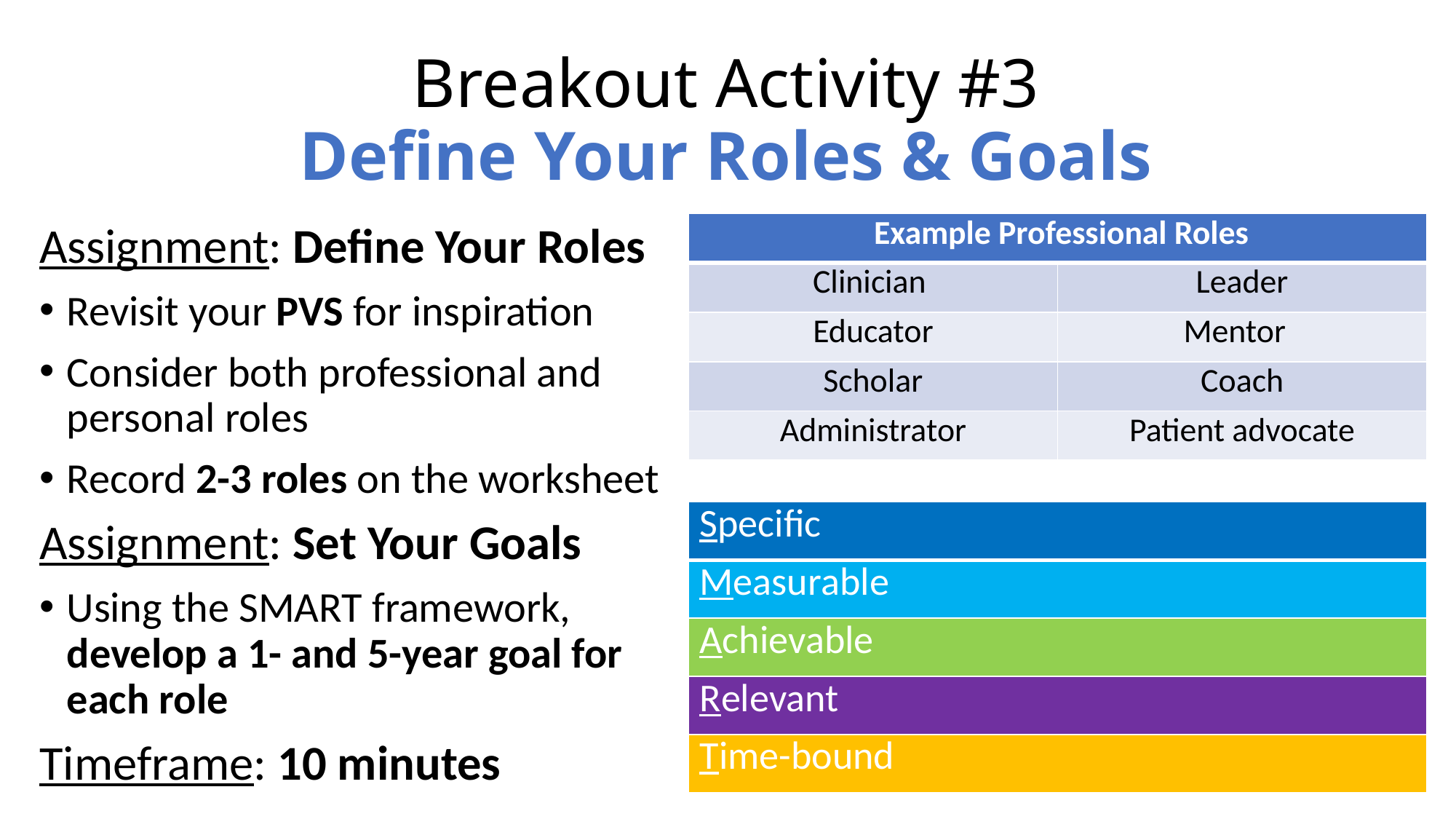

# Breakout Activity #3 Define Your Roles & Goals
| Example Professional Roles | |
| --- | --- |
| Clinician | Leader |
| Educator | Mentor |
| Scholar | Coach |
| Administrator | Patient advocate |
Assignment: Define Your Roles
Revisit your PVS for inspiration
Consider both professional and personal roles
Record 2-3 roles on the worksheet
Assignment: Set Your Goals
Using the SMART framework, develop a 1- and 5-year goal for each role
Timeframe: 10 minutes
| Specific |
| --- |
| Measurable |
| Achievable |
| Relevant |
| Time-bound |
28

## Slide 29
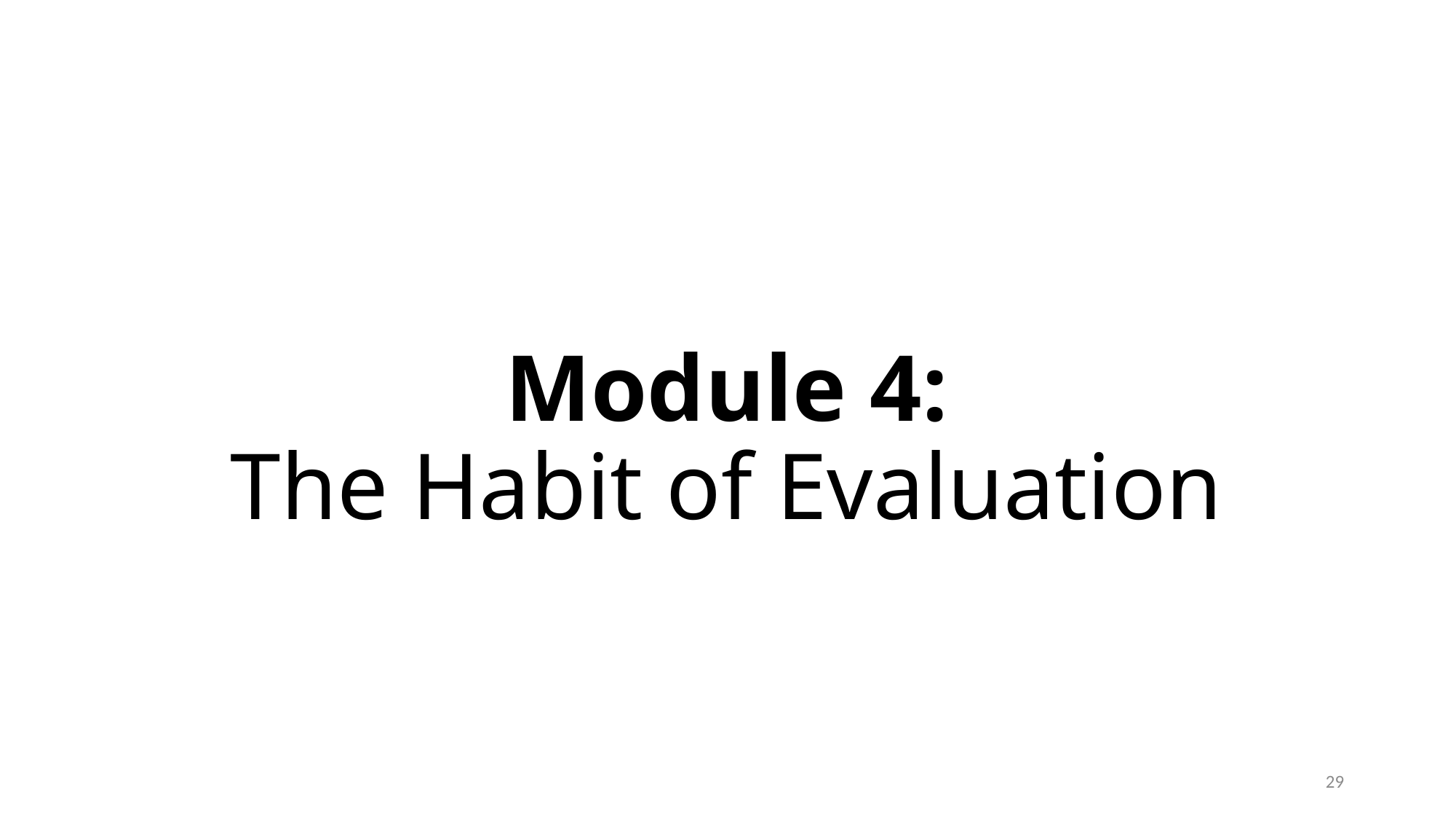

# Module 4: The Habit of Evaluation
29

## Slide 30
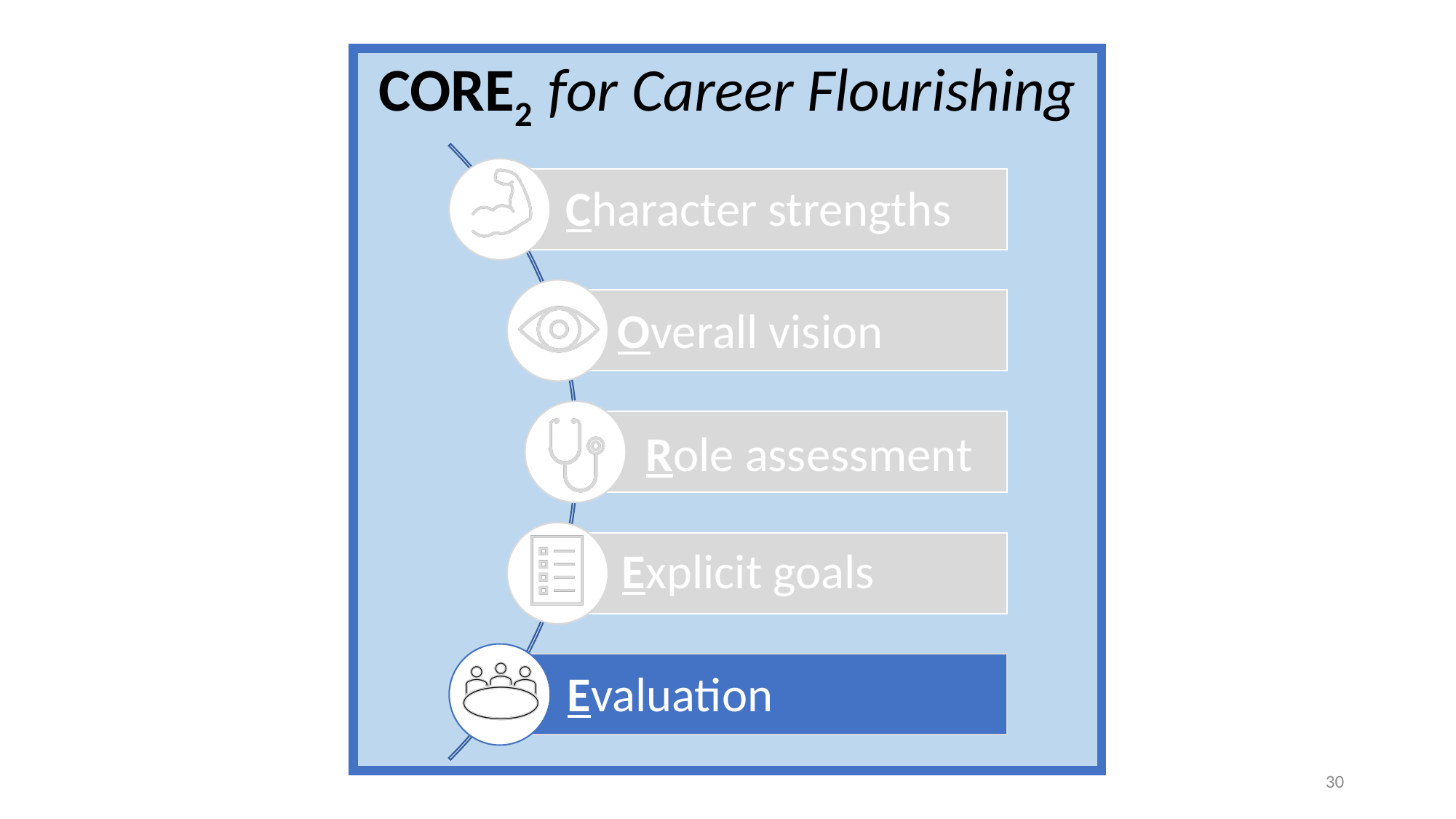

CORE2 for Career Flourishing
Character strengths
Overall vision
Role assessment
Explicit goals
Evaluation
30

## Slide 31
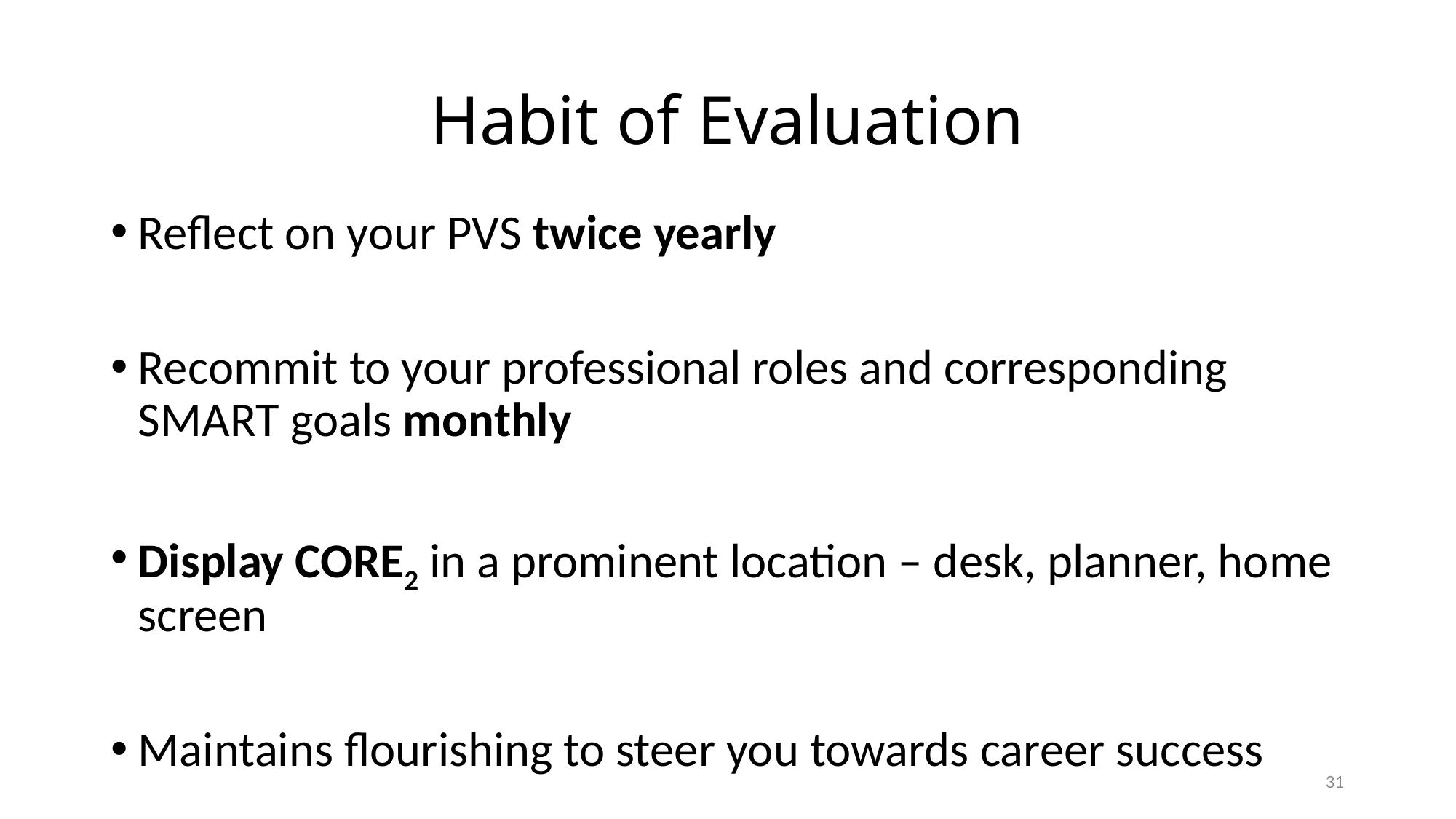

# Habit of Evaluation
Reflect on your PVS twice yearly
Recommit to your professional roles and corresponding SMART goals monthly
Display CORE2 in a prominent location – desk, planner, home screen
Maintains flourishing to steer you towards career success
31

## Slide 32
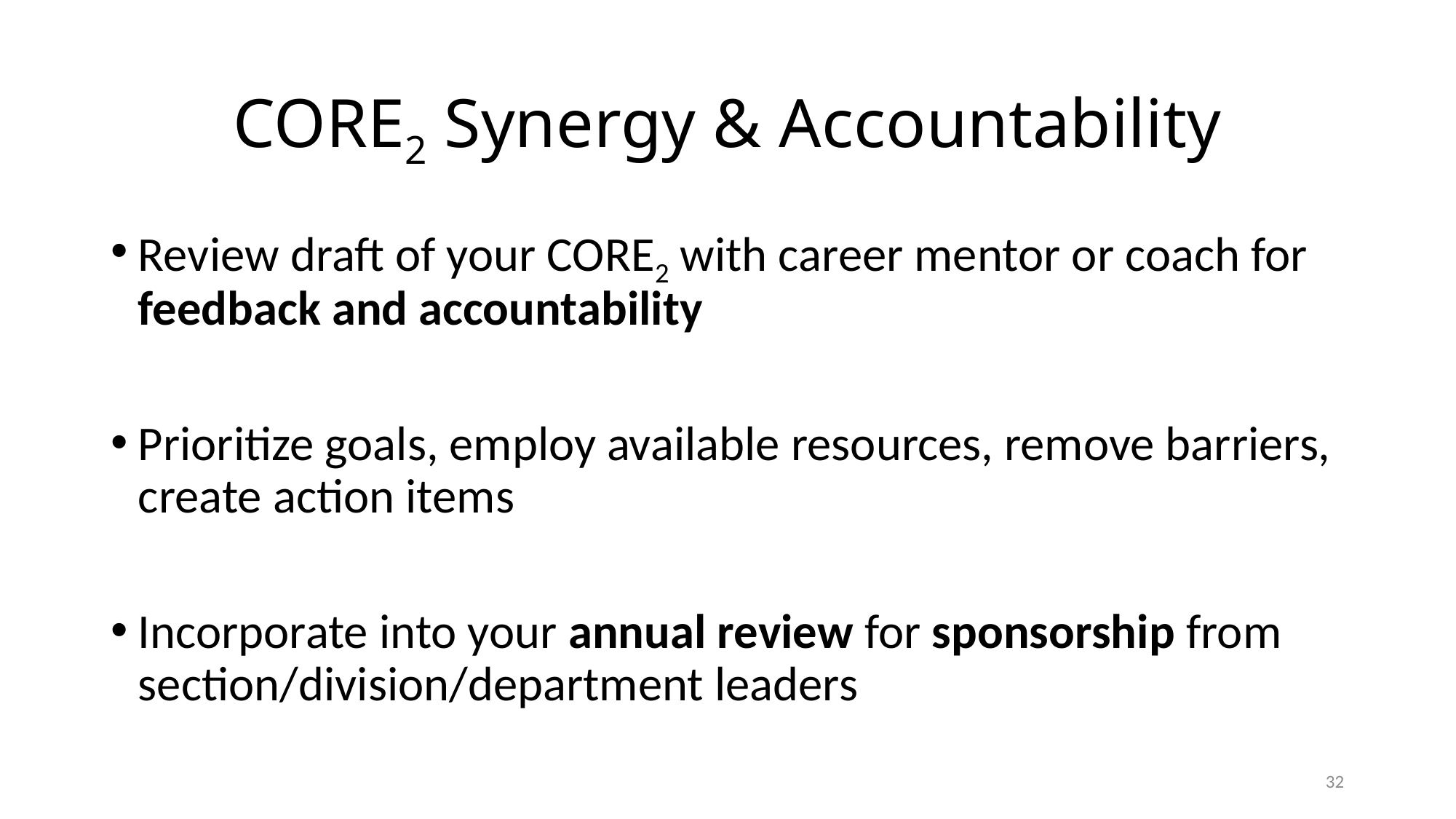

# CORE2 Synergy & Accountability
Review draft of your CORE2 with career mentor or coach for feedback and accountability
Prioritize goals, employ available resources, remove barriers, create action items
Incorporate into your annual review for sponsorship from section/division/department leaders
32

## Slide 33
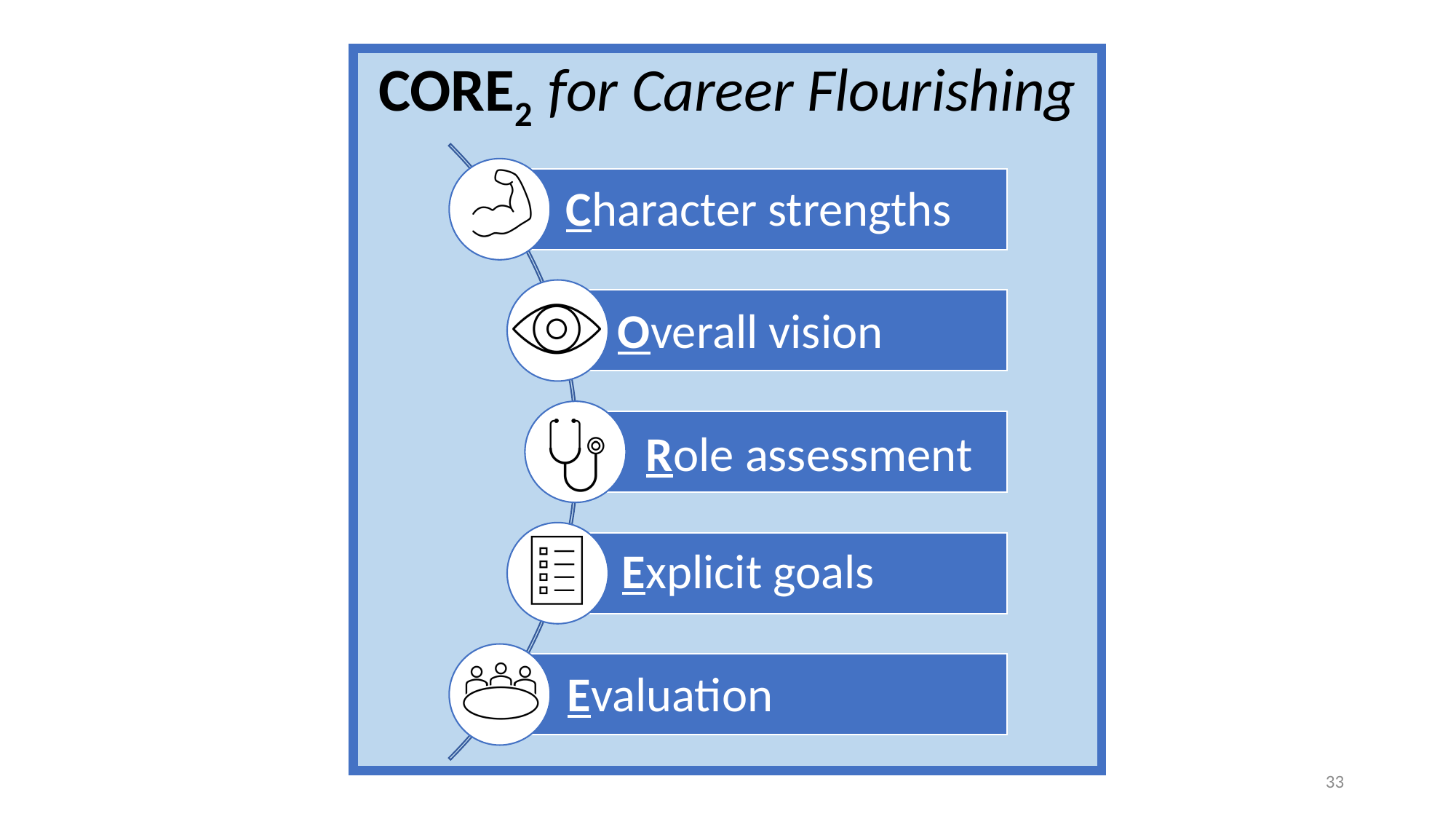

CORE2 for Career Flourishing
Character strengths
Overall vision
Role assessment
Explicit goals
Evaluation
33

## Slide 34
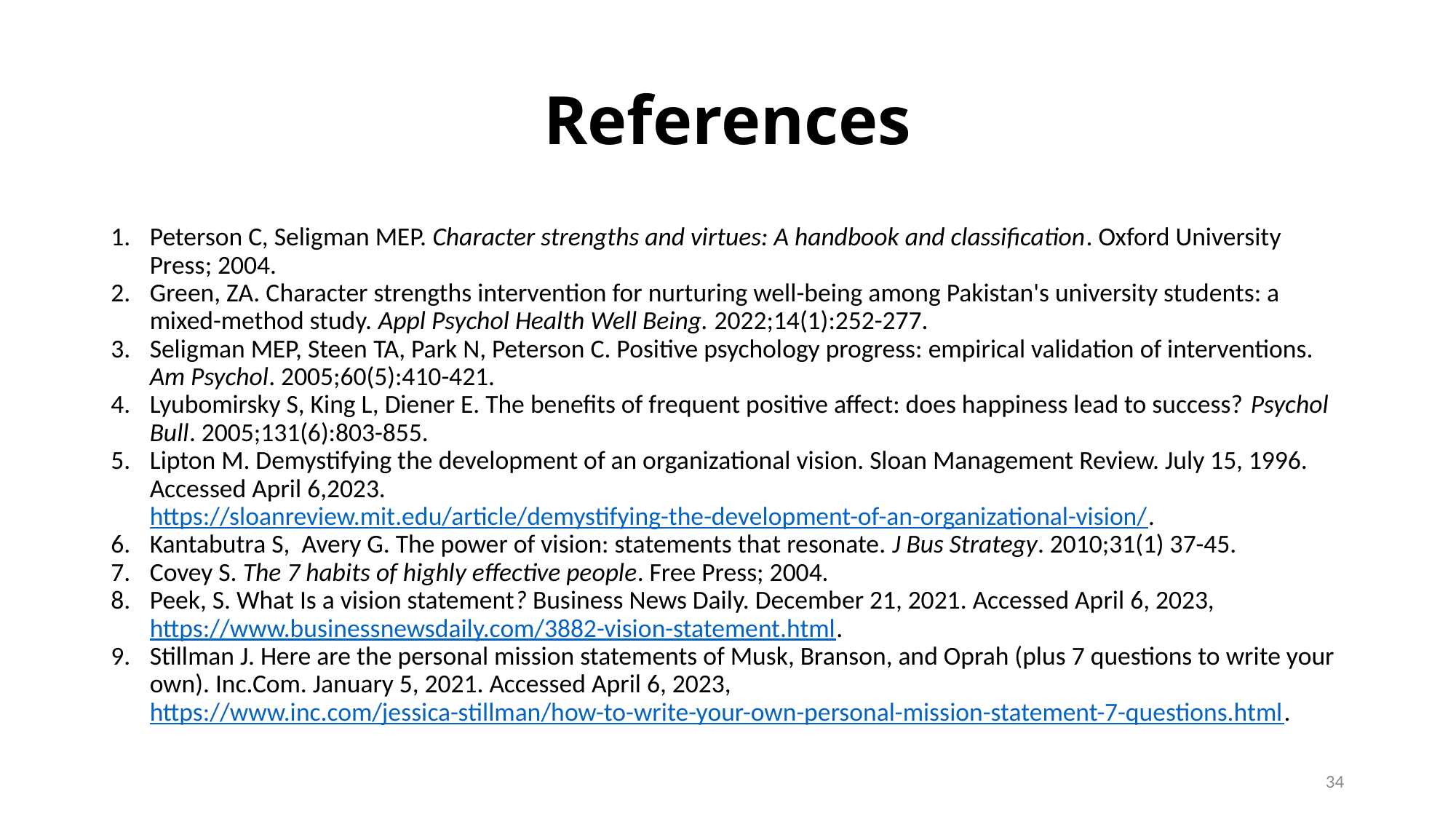

# References
Peterson C, Seligman MEP. Character strengths and virtues: A handbook and classification. Oxford University Press; 2004.
Green, ZA. Character strengths intervention for nurturing well-being among Pakistan's university students: a mixed-method study. Appl Psychol Health Well Being. 2022;14(1):252-277.
Seligman MEP, Steen TA, Park N, Peterson C. Positive psychology progress: empirical validation of interventions. Am Psychol. 2005;60(5):410-421.
Lyubomirsky S, King L, Diener E. The benefits of frequent positive affect: does happiness lead to success? Psychol Bull. 2005;131(6):803-855.
Lipton M. Demystifying the development of an organizational vision. Sloan Management Review. July 15, 1996. Accessed April 6,2023. https://sloanreview.mit.edu/article/demystifying-the-development-of-an-organizational-vision/.
Kantabutra S,  Avery G. The power of vision: statements that resonate. J Bus Strategy. 2010;31(1) 37-45.
Covey S. The 7 habits of highly effective people. Free Press; 2004.
Peek, S. What Is a vision statement? Business News Daily. December 21, 2021. Accessed April 6, 2023, https://www.businessnewsdaily.com/3882-vision-statement.html.
Stillman J. Here are the personal mission statements of Musk, Branson, and Oprah (plus 7 questions to write your own). Inc.Com. January 5, 2021. Accessed April 6, 2023, https://www.inc.com/jessica-stillman/how-to-write-your-own-personal-mission-statement-7-questions.html.
34
