# Supplementary material for: Develop Your CORE2 for Career Flourishing: A Career Development Workshop for Hospitalists
Source: MedEdPORTAL. 2024 Mar 15;20:11387. doi: 10.15766/mep_2374-8265.11387 (PMC10940547; doi:10.15766/mep_2374-8265.11387)
Supplement: Supplementary file 1 — Modules 1-4.pptxCharacter Strengths and Virtues Handout.docxParticipant Worksheet.docxGraphic Template.pptxFacilitator Guide.docxPresurvey.docxPostsurvey.docx [file mep_2374-8265.11387-s001.zip › D. Graphic Template.pptx]

## Slide 1
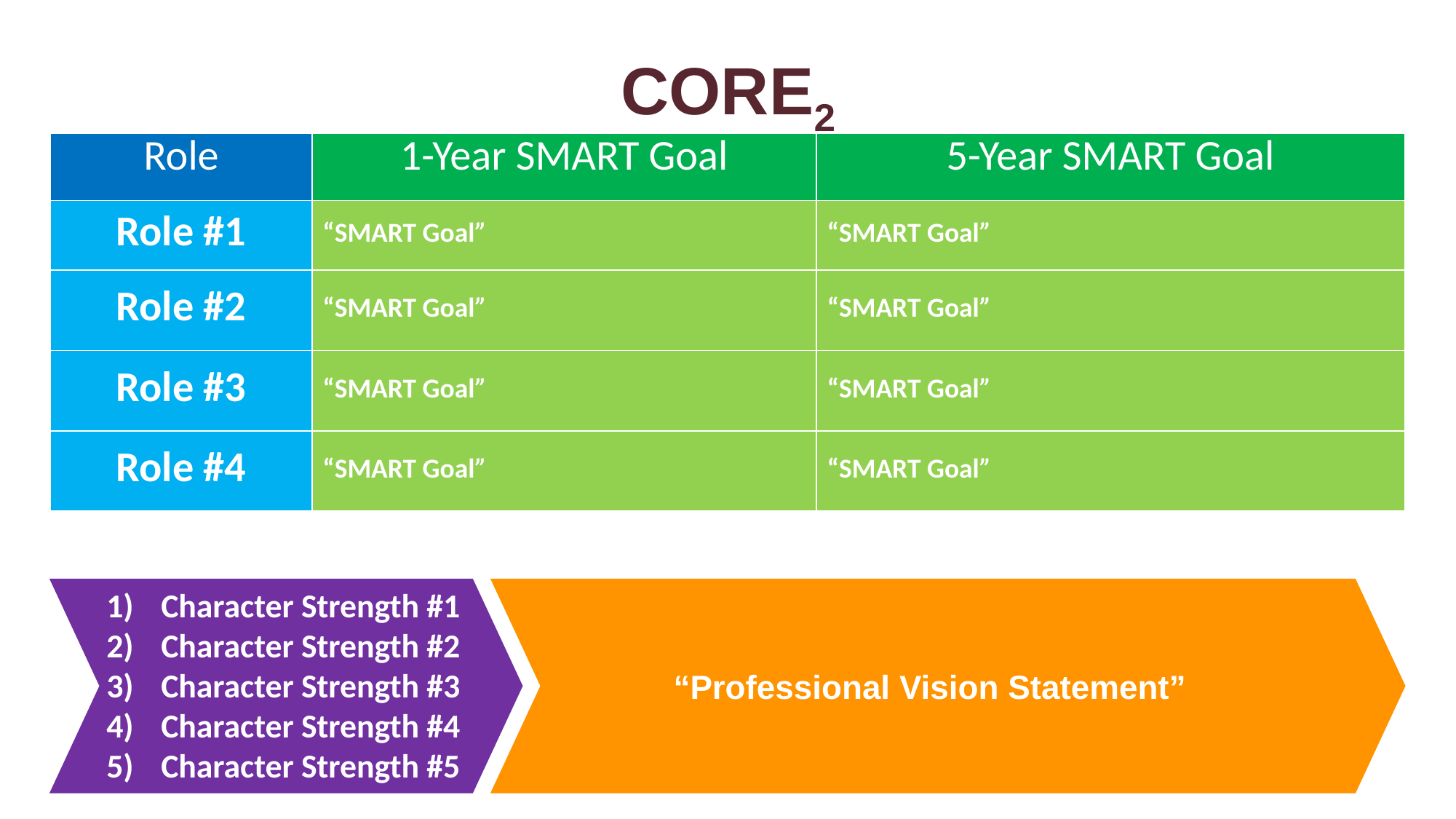

CORE2
| Role | 1-Year SMART Goal | 5-Year SMART Goal |
| --- | --- | --- |
| Role #1 | “SMART Goal” | “SMART Goal” |
| Role #2 | “SMART Goal” | “SMART Goal” |
| Role #3 | “SMART Goal” | “SMART Goal” |
| Role #4 | “SMART Goal” | “SMART Goal” |
Character Strength #1
Character Strength #2
Character Strength #3
Character Strength #4
Character Strength #5
“Professional Vision Statement”
